# Cyclodextrins tethered with oligolactides – green synthesis and structural assessment

**DOI:** 10.3762/bjoc.13.77

**Published:** 2017-04-26

**Authors:** Cristian Peptu, Mihaela Balan-Porcarasu, Alena Šišková, Ľudovít Škultéty, Jaroslav Mosnáček

**Affiliations:** 1 Polymer Institute of Slovak Academy of Sciences, Dúbravská cesta 9, 84541 Bratislava, Slovakia; 2 “Petru Poni” Institute of Macromolecular Chemistry, Alee Grigore Gica Voda 41A, 700487 Iasi, Romania; 3 Institute of Virology, Biomedical Research Center Slovak Academy of Sciences, Dúbravská cesta 9, 84541 Bratislava, Slovakia

**Keywords:** cyclodextrin, ESI, evaporative light scattering detection, liquid chromatography, L-lactide, MALDI, mass spectrometry, NMR

## Abstract

Biodegradable oligolactide derivatives based on α-, β- and γ-cyclodextrins (CDs) were synthesized by a green procedure in which CDs play the role of both the initiator and the catalyst. The synthetic procedure in which CDs and L-lactide (L-LA) are reacting in bulk at relatively high temperature of 110 °C was investigated considering the structural composition of the products. The obtained products were thoroughly characterized via mass spectrometry methods with soft ionization like matrix-assisted laser desorption ionization (MALDI) and electrospray ionization (ESI). Liquid chromatography (LC) separation with evaporative light scattering detection (ELSD) and NMR analysis were employed in order to elucidate the structural profiles of the obtained mixtures. The results clearly demonstrate that the cyclodextrins were tethered with more than one short oligolactate chain per CD molecule, predominantly at the methylene group, through ring opening of L-LA initiated by primary OH groups.

## Introduction

Cyclodextrin derivatives are increasingly important and their variety is dictated by the wide range of applications in which these compounds are employed with preponderance in the pharmaceutical field [1–2]. The employed strategies for the modification with small molecular weight compounds are taking advantage of the different reactivity of the hydroxy groups in 2, 3 or 6 position, thus allowing selective modifications [3]. While modified CDs with low molecular weight substituents, such as methyl, (2-hydroxy)propyl, sulfobutyl, etc. are already available as commercial products, the modification with polymers is still under development [4–5]. So far, several polymerization reactions were used for CD modification, including free radical polymerization, reversible-deactivation radical polymerizations [5] as well as ring opening polymerizations (ROP) of cyclic esters [6], oxiranes [7] and oxazolines [8]. However, the ROP of cyclic esters should also be considered as a method of producing polymer-modified CDs with some particular features, such as possibility of employing green polymerization procedures and availability of renewable monomers like cyclic esters. The methods published so far for polymerization of cyclic esters initiated by cyclodextrins employed catalysts commonly used in ROP, such as Sn-octoate [9–11] or amine-based organic catalysts [12], resulting in star polymers with a more or less well defined structure. CD functional polylactides have been prepared using different catalytic systems with good results in synthesis of star polymers with relatively high molecular weight and low polydispersity, by the “core first” method. Polymerization of L-lactide was performed by anionic ROP initiated by potassium alkoxides of α-CD partially modified with trimethylsilazane [13]. The L- or DL-lactide were polymerized in the presence of organocatalysts like 4-dimethylaminopyridine [12] using β-CD and modified CD (β-CD-(OBn)_19_(OH)_2_) as initiators. Normand et al. [14] applied a similar approach as Zinck and co-workers [12] in order to prepare CD-containing polymers while simplifying the complexity induced by multifunctional initiator through partial benzylation of the β-CD resulting in a CD-diol. Also, the ROP of D,L-LA catalyzed by 4-dimethylaminopyridine and initiated by all 21 OH groups of β-CD was employed by Xu et al. [15].

However, the above mentioned methods were using CDs only as a scaffold for growing star polymers with properties belonging more to the class of polymers and, in consequence, the CD core influenced the properties of the final product in a small proportion. Thus, these polymers do not differ significantly from other star polymers with different core and similar number of arms. The modification of CDs with a reduced amount of monomer units results in CD-oligomer materials which still keep an important property of the starting CDs, like their inclusion ability [16]. The CD-oligoester conjugate, compounds with a relatively low content of oligoester components were first prepared by a totally green procedure by Harada and co-workers [17]. They succeeded to polymerize a series of cyclic esters including β-butyrolactone (BL), δ-valerolactone (VL) and ε-caprolactone (CL). A recent work published by Galia et al. [18] brings new insights on the effect of pressure on bulk polymerization of CL initiated by β-CD. The lactides polymerization (L-LA, D-LA and DL-LA) was also attempted [19–20] but with less success, as compared with previously mentioned cyclic esters, possibly, due to the fact that the LA monomers (especially DL-LA) are solid in the range of temperature applied during the reaction. Attempts to resolve the reactants mixing problem were made by using δ-valerolactone (VL) as dispersion environment for the lactides. A better overall conversion and increased molecular weights were observed but the authors did not asses their products whether these were CD-VL, CD-LA or CD-VL/LA covalent conjugates [19–20]. The results presented by the Harada group generally evaluated the CD-oligoester samples by matrix-assisted laser desorption ionization mass spectrometry (MALDI–MS) and nuclear magnetic resonance (NMR) spectroscopy, however, in case of CD-oligolactides no such structural proofs were presented. Later, the LA was polymerized in dimethylformamide (DMF) solution to result in well-defined and homogenous β-CD functionalized with oligolactides as demonstrated by electrospray mass spectrometry (ESI–MS) and NMR spectroscopy [16].

Herein we analyze the products resulted from a green synthetic approach and clarify the structure of the obtained products. Therefore, we present an alternative route of bulk polymerization of L-LA, in the presence of α-, β- and γ-cyclodextrins, in melt system and the obtained products are thoroughly evaluated by various analytical methods, such as mass spectrometry, NMR spectroscopy and reversed-phase liquid chromatography. Results for particular cyclodextrins (α-, β- and γ-) are compared in order to understand the influence of type of CD on the course of modification.

## Results and Discussion

The properties of cyclodextrin-oligoester covalent conjugates, situated at the border of low and high molecular weight compounds can be influenced by both the carbohydrate and the oligoester components. The properties of such materials like solubility, miscibility with other materials, inclusion capacity or even degradability in different environments will depend on their structure which is therefore crucially important to be uncovered at the molecular level.

Nevertheless, to completely understand the related structural issues is a difficult task due to the level of complexity of such products. One particular problem is arising from the presence of multiple reactive points, i.e., OH groups, in the CD structure. On any given CD molecule there are three types of OH groups with their own specificities, like OH6 groups are the most accessible for bulky substituents, the OH2 are the most acidic and OH3 groups are the least reactive [3]. This heterogeneity may favor a specific attachment of cyclic esters to the CDs but also can lead to a certain non-uniformity or isomeric distribution (dispersity) of the modified CDs. Another source of sample dispersity is the molecular weight dispersity commonly encountered in synthetic polymers. Moreover, the presence of multiple initiating sites may favor the formation of star-like oligomers. A particular issue, still under debate, is related to the possibility that in spite of multiple reactive points, namely the OH groups, which can be involved in ring opening of cyclic esters, one cyclodextrin molecule initiates a single polymer chain [17,20]. This fact was justified by the steric hindrance created by a first covalent attachment of a cyclic ester to the CD, which does not allow the growth of another chain from a single CD molecule. However, we already showed by MS in combination with NMR spectroscopy that, in case of β-BL bulk and solution polymerization in the presence of (−)-sparteine as organic catalyst, multiple chain attachment was possible in spite of possible steric hindrance [21–22]. Considering all that, a cautious and deep characterization should be performed in order to fully describe such systems. We showed that understanding of molecular level structural features of such complex mixtures (free CDs, homopolymers, CD-oligoesters) have to take into consideration the mass spectrometry methods in direct correlation with the NMR spectroscopy. Liquid chromatography and tandem MS fragmentation studies [21–23] are also important additions in deeper structural characterization of CD-oligoester conjugates.

**Scheme 1:**
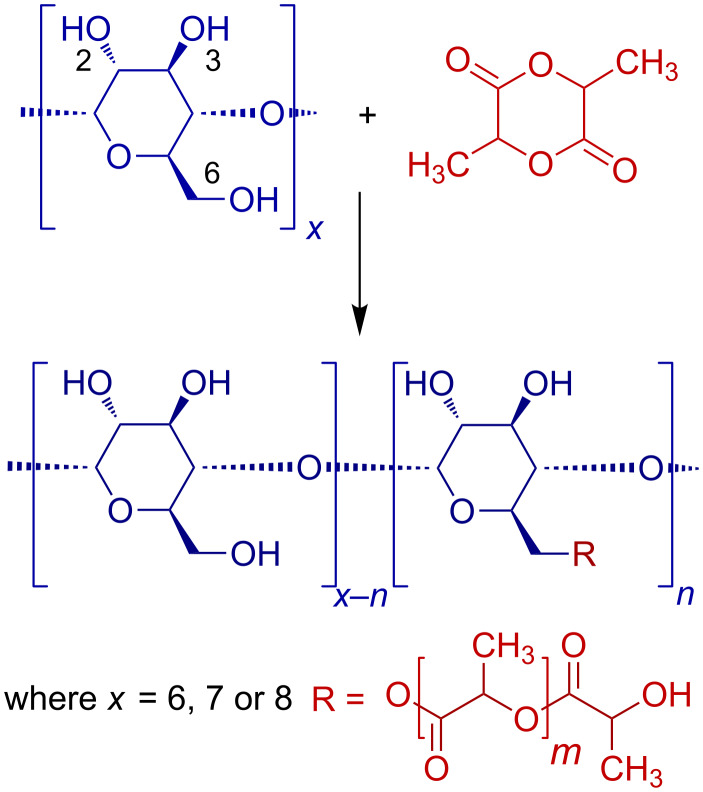
Ring opening of L-LA in the presence of cyclodextrins.

The L-LA was reacted with α-, β- and γ-CD (Scheme 1) in bulk at 110 °C in order to ensure a good dispersion of reactants. The molar ratio between L-LA and OH groups of CDs was kept to a value of 5 for all types of CDs (Table 1) and the reaction was stopped after 72 hours. Although the cyclodextrins were only dispersed in the molten monomer the magnetic stirring insured a good dispersion of the reactants. In previous studies [19], the molar ratio between CD and the total monomer amounts introduced in the reaction was about 1/5 which even, in conditions of melted monomer, would not ensure a good mixture of reactants and therefore the reaction would be considered as a heterogeneous system with all disadvantages resulting from this.

**Table 1:** Characterization of CD-LA products.

Sample	OH/LA molar ratio	CD/LA molar ratio	% of free CD^a^	Weight % of F1 fraction	*M*_n_/*Đ*^b^	MS analysis of F1^c^	MS analysis of F2^c^

α-CD-LA	1/5	1/90	10	2	1600/1.75	1192/1.36	2798/12.52
β-CD-LA	1/5	1/105	10.2	5.5	2400/1.68	1296/0.96	3725/17.83
γ-CD-LA	1/5	1/120	30.5	9	1700/1.52	1878/3.87	3264/13.50

^a^Fraction determined by integration of peaks from ELSD after application of reversed-phase chromatography. Calculated as % free CD = total area peaks/area of free CD in LC ELSD chromatograms of raw reaction mixtures. ^b^Determined by GPC of F2. ^c^Average molecular weight and number of dilactate monomer units for one cyclodextrin (α-, β- or γ-CD) molecule determined by MALDI–MS [average mass = sum(*m*_i_*n*_i_)/sum(*n*_i_), where *m*_i_ = the *m*/*z* value of all peaks of CD derivatives from the MALDI–MS spectrum and *n*_i_ = the relative intensities of the corresponding MS peaks; average number of monomer units = (average mass–mass of corresponding CD-mass of Na cation)/mass of dilactate monomer unit]

The assessment of reaction products, obtained under the above mentioned conditions, was rather complicated because the products were not fully soluble either in water or in organic solvents like THF or ACN. In principle, the resulted mixture contained free CD, CD modified with polylactide units (CD-LA), polylactide (PLA) homopolymers and unreacted L-LA monomer. The ^1^H NMR of crude reaction mixture showed that monomer conversion after 72 h was slightly over 5% for all CD initiators, which is in good correlation with conversion described by other authors for similar systems [18–19].

**Figure 1:**
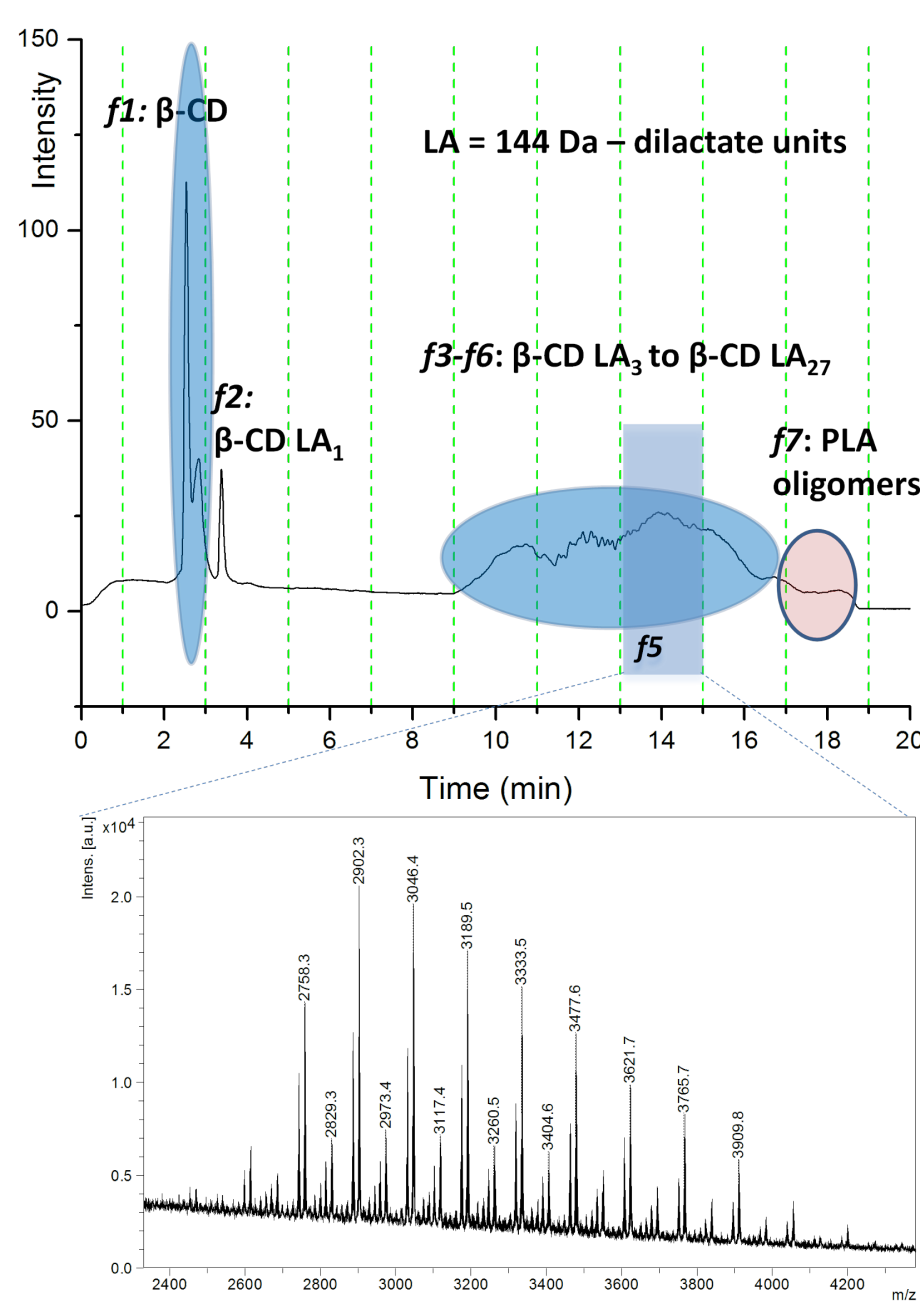
(a) ELSD chromatogram of crude β-CD-LA reaction mixture and (b) MALDI–MS spectrum of fraction *f*5.

First we performed the reversed-phase liquid chromatography (LC) separation of the crude reaction mixture with evaporative light scattering detection (ELSD) for all CD-LA products. The chromatogram depicted in Figure 1a contains two distinct groups of chromatographic peaks of β-CD-LA reaction mixture. The first group appear at low elution time (from 2 to 4 min) and has increased water solubility, suggesting the presence of unmodified CD and CDs with low substitution degree. The second group, eluted from 9 to 18 minutes, contained modified CDs and PLA oligomers. The overlaid LC chromatograms for crude α-, β- and γ-CD-LA products are shown in Supporting Information File 1, Figure S1. The nature of the separated compounds was partially confirmed by "off line" MALDI–MS measurements of the eluted fractions. Full MALDI–MS identification of all collected LC fractions for α-, β- and γ-CD-LA and LC ELSD chromatograms are shown in Supporting Information File 1, Figures S3–S21.

Based on the MALDI–MS identification, the main chromatographic peak in Figure 1a has been assigned to free β-CD (fraction *f*1) while the other eluted species are β-CD-LA having from 1 to 27 monomer units (dilactate), fractions *f*2–*f*6 (elution times: *f*2 – 3–5 min, *f*3 – 9–11 min*, f*4 *–* 11–13 min*, f*5 – 13–15 min, *f*6 – 15–17 min and *f*7 – 17–19 min). In Figure 1b the MALDI–MS identification of chromatographic peaks for the β-CD-LA sample, chromatographic fraction *f*5 eluted between 13 and 15 minutes, is exemplified. In the MS spectrum, the structural assignment was performed for the respective *m*/*z* values using the following equation: *m*/*z* = 1134 (β-CD) + *n* * 144 (LA) + 39 (K). The main peaks of the considered series were adducts of K^+^, but adducts with Na^+^ were also identified (Δ*m*/*z* = −16). Considering the lactate (72 Da) as a monomer unit, the oligomers with odd number of monomer units could be observed as well. These oligomer species with odd number of lactates resulted from intramolecular and/or intermolecular transesterification reactions and are in lower amount, when compared with the oligomers containing even number of monomers. This signifies that the predominance of transesterification is rather low in the employed reaction conditions. The presented spectrum was measured directly from the HPLC collected fraction with rather low concentration of products, thus resulting in a poor spectrum quality.

The *f*7 fraction (Figure 1 **–** LC–ELSD chromatogram and Figure S9 (Supporting Information File 1) **–** MALDI–MS spectrum) contained only PLA oligomers having from 16 to 28 lactate monomer units. However, all fractions from *f*3 to *f*7 may contain PLA homopolymers which are co-eluted with the CD-LA product (vide infra LC–ESI–MS characterization). The LC–MALDI–MS allowed estimating the maximum number of monomer units per CD molecule for each of the α-, β- and γ-CD systems. It may be observed that the highest number of L-LA monomer units were grafted on β-CD (27 dilactate units) while for α-CD and γ-CD were obtained maximum values of 18 and 19 dilactate units, respectively. Thus, β-CD seems to have the best activity in ring opening of L-LA.

The ELS detection allowed a quantitative evaluation of the reaction mixture content (Table 1). Thus, it may be observed that similar values of the relative content of free CD were obtained for α- and β-CD while, in case of γ-CD, the relative amount of free CD in the reaction mixture was three times higher. Therefore we may hypothesize that for similar reaction conditions the γ-CD is less active in the ring opening of L-LA. The calculation of the relative amount of free CD was performed by integration of chromatographic peaks corresponding to free CD and to modified CD in ELSD chromatograms. However, this estimation was made under the reserve that CD-LA mixtures eluted after 8 min may contain also PLA oligomer species initiated by the presence of residual water in the reaction system.

We chose "off line" confirmation of the eluted species by MALDI–MS over other available methods, as LC with "on line" ESI–MS detection has problems in the analysis of polymer species related to the formation of multicharged species, thus creating difficulties in the spectra interpretation, especially in the case of polydisperse oligomer mixtures [24–25]. However, the presence of PLA oligomers was clearly confirmed by LC–ESI–MS, which has a better sensitivity for linear low-molecular-weight oligolactides. In the case of MALDI–MS identification, the presence of the substance used as matrix prevents accurate observation of the region below *m*/*z* = 600 and therefore only PLA oligomers with a mass higher than this threshold were observed (e.g., *f*7 for β-CD-LA). The LC separation with ESI–MS detection, performed only for β-CD-LA sample showed clearly that PLA oligomers are co-eluted with the β-CD-LA products. The presence of PLA oligomers can be justified by water initiation of L-lactide ring opening. The relative amount of homooligolactide species is highly exaggerated in the presented ESI chromatogram (Supporting Information File 1, Figure S22) due to mass discrimination of low-molecular-weight compounds (PLA) against co-eluting higher molecular-weight compounds (β-CD-LA) under electrospray conditions. This mass discrimination together with the formation of multiple charged species (example of ESI–MS spectrum of double and triple charged β-CD-LA is provided in Supporting Information File 1, Figure S23) is actually hindering a comprehensive evaluation of these CD-LA samples by LC with ESI–MS detection [26].

The presence of water in the reaction mixture may be debated, however, it was previously stated that, even with thorough procedures of water removal from cyclodextrin, water traces are still present [7]. Moreover, it was shown recently [18] that water is actually improving the overall process in case of bulk polymerization of ε-caprolactone (CL) under conditions of increased pressure. In the above mentioned study, β-CD modified with CL was characterized by MALDI–MS. Mixtures of PCL hompolymers and CD-caprolactone modified conjugates were evaluated in view of the products relative content only based on the MALDI–MS spectra. Generally, compounds with different ionization efficiency in MALDI–MS will give different MS response or intensity of the corresponding MS peaks [27] which does not allow a precise quantitative measurement. In our current study we do not attempt such comparison considering that the relative amount of modified CD and homopolyester obtained by such calculation would be highly biased.

Previously, we showed that LC–ESI–MS evaluation is possible for CD-(3-OH butyrate) conjugates taking into consideration only the MS peaks of structurally similar compounds with low molecular weight and molecular weight dispersity, i.e., CDs tethered with an average of 12 monomer units [21]. In the work of Shen et al. [16], where homogenous CD-LA conjugates were evaluated by ESI–MS with direct sample injection, the average number of dilactate monomer units per CD molecule was around 3.5 which is clearly lower than in the case of CD-LA products described here.

The ESI–MS evaluation proved to be difficult and it was obvious that a different mass spectrometry evaluation of CD-LA products would be needed. In the previous papers [17] it was stated that CD-oligoester products may be separated from the reaction mixture by dissolution in DMF and precipitation into excess of dry THF in order to remove the unreacted CD. The procedure applied here resulted in two fractions for each sample, the THF insoluble (F1) and THF soluble (F2). This fractionation allowed analysis by MALDI–MS of the CD fraction with higher substitution degree.

**Figure 2:**
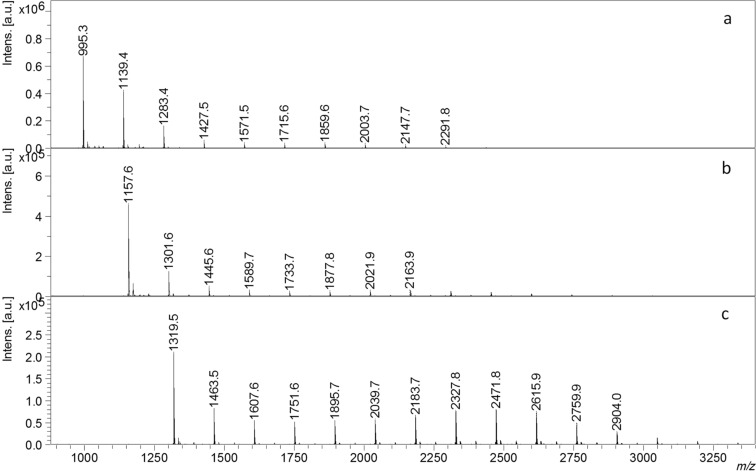
MALDI–MS spectra of the fractionated α-, β- and γ-CD-LA products – fractions precipitated in THF: (a) α-CD-LA fraction F1A, (b) β-CD-LA fraction F1B and (c) γ-CD-LA fraction F1C.

**Figure 3:**
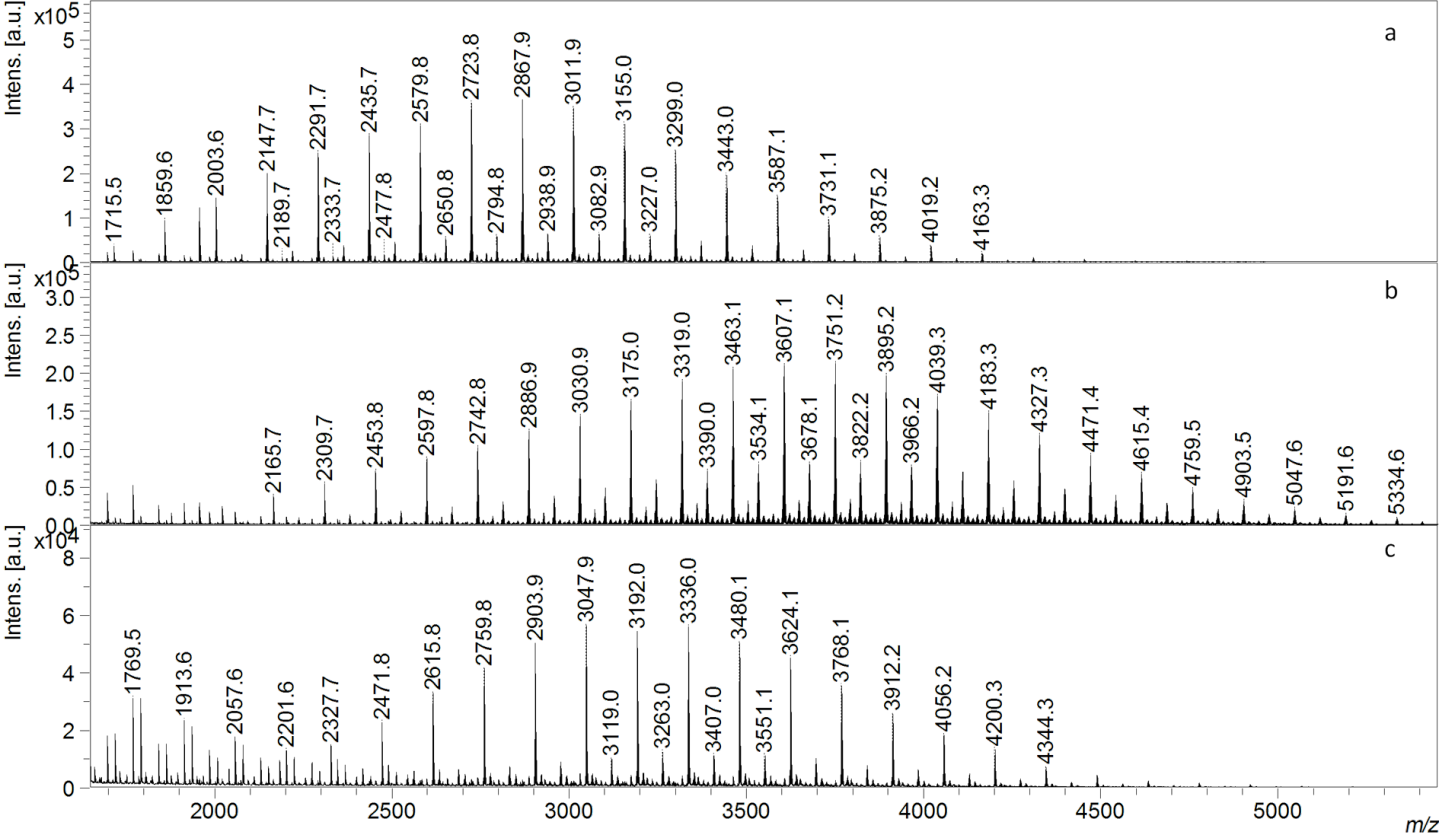
MALDI–MS spectra of the fractionated α-, β- and γ-CD-LA products – fractions soluble in THF: (a) α-CD-LA fraction F2A, (b) β-CD-LA fraction F2B and (c) γ-CD-LA fraction F2C.

The fractions separated (Experimental section – vide infra) in case of α-CD-LA (F1A and F2A) are both described using the MALDI–MS. The MS spectrum of the F1A fraction, in Figure 2a**,** revealed the presence of free α-CD and α-CD-LA oligomers having from 1 to 8 dilactate monomer units. The general formula used for the calculation of the *m*/*z* values corresponding to the assigned structures was *m*/*z* = 972 (α-CD) + *n* * 144 (LA) + 23 (Na). The most intense peaks corresponded to the species having from 0 to 2 monomer units. Thus, the fractionation procedure led to the loss of CD conjugates with a low number of LA monomer units. The THF soluble fraction, F2A, (Figure 3a) was composed of α-CD-LA oligomers having from 5 to 22 dilactate units. The average *M*_n_ calculated from the relative intensities of the MS peaks was 2800 g/mol and corresponded to an average degree of polymerization of 13 dilactate monomer units. In the F2A spectrum a second series of peaks situated at 72 Da difference from the members of the main series may be also be observed, which correspond to α-CD-LA species with an odd number of lactate monomer units. These species could be formed as a result of transesterification reactions (intra- or intermolecular), which may occur in the melt reaction system. The presence of PLA homopolymers was also observed, their corresponding *m*/*z* being calculated by the following equation: *m*/*z* = 18 (H_2_O) + *n* * 144 (LA) +23 (Na)].

In a similar manner, the fractions collected in the case of β- and γ-CD-LA were analyzed by MALDI–MS (Figure 2b and Figure 3b). For β-CD-LA the formula used for the assignment of the MS peaks was *m*/*z* = 1134 (β-CD) + *n* * 144 (LA) +23 (Na). Based on these calculations, we could remark that also in the case of the β-CD-LA fractionation some part of the species with lower polymerization degree were precipitated together with free CD (β-CD-LA F1B fraction had dilactate monomer units from *n* = 0 to *n* = 10 – Figure 2b). On the other hand, the β-CD-LA fraction F2B (Figure 3b) had a number of monomer units ranging from 6 to 28 dilactate units. The *M*_n_ inferred from the MS spectrum had a value of 3800 g/mol corresponding to approximately 18 dilactate monomer units. The peaks from the species with an odd number of lactate monomer units formed by transesterification reactions (Δ*m*/*z* = 72 Da from the main series) were also present in the spectra. In addition, in the lower region of the MALDI spectrum some peaks corresponding to PLA homopolymers in the lower mass region of the spectrum could also be observed.

The γ-CD-LA fractions, γ-CD-LA F1C (precipitated in THF) and γ-CD-LA F2C (soluble in THF), presented in Figure 2c and Figure 3c, could be described based on the MS spectra by using the following equation: *m*/*z* = 1296 (γ-CD) +*n* * 144 (LA) +23 (Na). The F1C fraction (Figure 2c) was more abundant in CD-LA species having from *n* = 0 to *n* = 13 dilactate units. The F2C fraction (Figure 3c) contains γ-CD-LA species having from 7 to 21 dilactate monomer units with an average of 14 monomer units corresponding to an *M*_n_ = 3300 g/mol. Also, PLA homopolymers having up to 9 dilactate monomer units were present in this sample. The presence of PLA oligomer species in all the analyzed F2 fractions was also confirmed by GPC of all F2 fractions. It was observed that in each case the GPC curves were bimodal with dispersity of around 1.6 (Table 1).

Exact evaluation of the resulted products was prevented due to the complexity of the mixtures containing CD-LA, PLA and L-LA. The MALDI analysis of F1 fractions (Figure 2) showed that the number of lactate units attached to the CD is different according to the influence of the type of cyclodextrin. The F1C fraction had more lactate units as compared with F1A and F1B fractions. This could be explained by a more significant contribution of the γ-CD part, which is bigger than other CD homologues (8 glycoside units), to the lower solubility in THF resulting in precipitation of γ-CD-LA species with higher content of dilactate units. This fact is also reflected by the different weight percentage of F1 fractions. The α-CD-LA F1 was on 2%, β-CD-LA F1 was 5.5% and γ-CD-LA-F1 was 9% weight from total reaction mixture (Table 1).

Thus, the proposed fractionation of the CD-oligoester samples did not provide proper purification and a significant part of the sample can be lost by precipitation. The purification procedure applied in the work of Shen et al. [16] was based on a precipitation of CD-LA, synthesized in DMF, in excess of dry ether and subsequent washing of precipitate with acetone. However, this also can lead to the loss of fraction with high content of lactate units. Nevertheless, the reaction in DMF allowed the isolation of a homogenous product, with low polymerization degree, useful for preparation of inclusion complexes. However, for characterization purposes this sample fractionation is useful as long as both fractions are analyzed. MALDI spectra of F2 fractions (Figure 3) are clearly showing that the average number of monomer units per CD (Table 1) is the highest for the β-CD initiated reaction while α- and γ-CD gave almost similar results. Thus, we may infer that β-CD is the best fit for this reaction system. Also, if LC with ELSD is taken into consideration (free CD vs CD-LA) we may state that free CD is in the highest amount for γ-CD while for α- and β-CD this ratio is similar. Therefore, the performance of different CDs in L-LA polymerization (in current reaction conditions) is decreasing in the order β-CD > α-CD > γ-CD.

The structural analysis aimed the following targets: to determine the substitution degrees for CD based products; the substitution site on the glycosidic rings (C2, C3 and/or C6) and the average length of the oligolactide sequences. So far, the MALDI–MS measurement of the fractionated reaction mixtures allowed the observation of the average number of monomer units per CD molecule for the respective fractions. In order to elucidate the other structural issues NMR spectroscopy has been employed.

**Figure 4:**
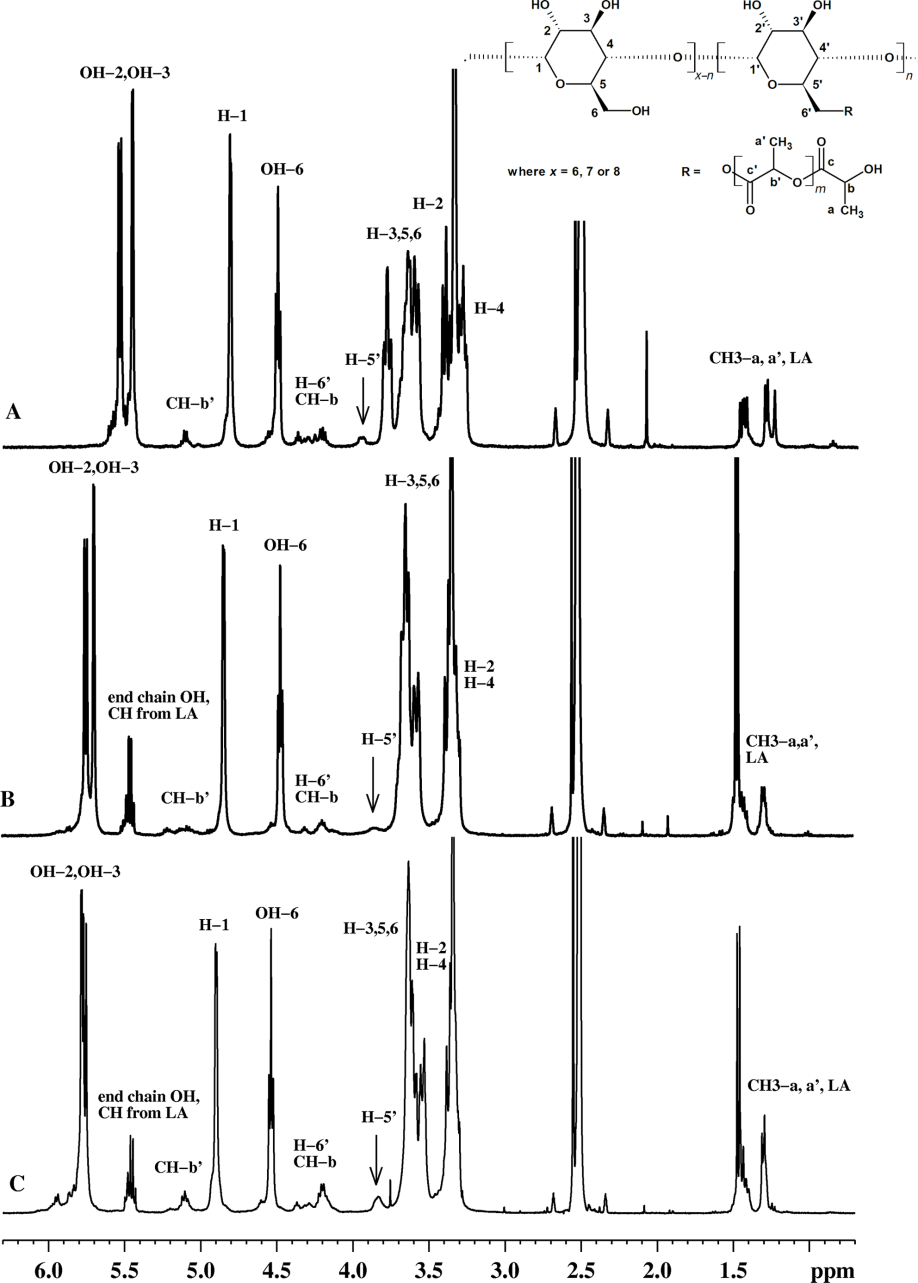
^1^H NMR spectrum of fractions precipitated in THF of (A) α-CD-LA, (B) β-CD-LA and (C) γ-CD-LA.

First, the ^1^H NMR spectroscopy analysis (Figure 4) was performed for the precipitated fractions (F1). These fractions had a lower content of L-lactide derived moieties, i.e., a high amount of free cyclodextrins and small amounts of functionalized CD-LA and L-lactide monomer. All samples showed peaks for the unreacted L-lactide and separated peaks for the protons of the substituted glucopyranose units. In the ^1^H NMR spectra the substitution pattern of the glycoside rings of cyclodextrins can be followed by comparing the integral for the anomeric proton, H1, with the integrals for the OH groups which must have a 1:1:1:1 ratio for the unreacted cyclodextrin. Upon substitution of one or more OH groups the integration ratio becomes unbalanced. In the case of the F1 fractions of α-CD-LA, β-CD-LA and γ-CD-LA we observed that this ratio is slightly unbalanced as some of the OH6 groups were esterified (Supporting Information File 1, Figures S24–S26). However, in the case of the β-CD-LA F2 fraction (Supporting Information File 1, Figure S27), the substitution degree was increased. An exact quantitation is prevented due to NMR peaks overlapping.

Generally, in the ^1^H NMR spectrum of the F2 fraction, the peaks corresponding to the cyclodextrin protons are broadened and the intensity of the signal corresponding to OH6 is flattened (almost not present) while those corresponding to OH2 and OH3 are still present but also broadened, with a ratio towards H1 proton close to 1:0.9, demonstrating that the CDs are substituted predominantly at C6. The analysis of the β-CD-LA F2 fraction was repeated at different time intervals in order to prevent errors in integral ratio calculations caused by a slow H/D exchange between the OH groups and DMSO-*d*_6_. Even though, these observations do not exclude some low degree of substitution of OH2 and OH3 groups, a certain tendency of selective substitution at C6 was confirmed, similar to the results obtained by Shen et al. [16], using DMF as solvent and possibly catalyst.

The covalent binding of the substituent induced a significant downfield shift for the peaks of the H6’ protons bound to the esterified carbon and also to the neighboring H5’ proton. These peaks, at about 4.2–4.3 ppm (H6’) and 3.8 ppm (H5’) were assigned by correlating the data obtained from ^1^H NMR, ^13^C NMR, DEPT135-NMR and 2D NMR spectroscopy (Supporting Information File 1, Figures S24–S31).

**Figure 5:**
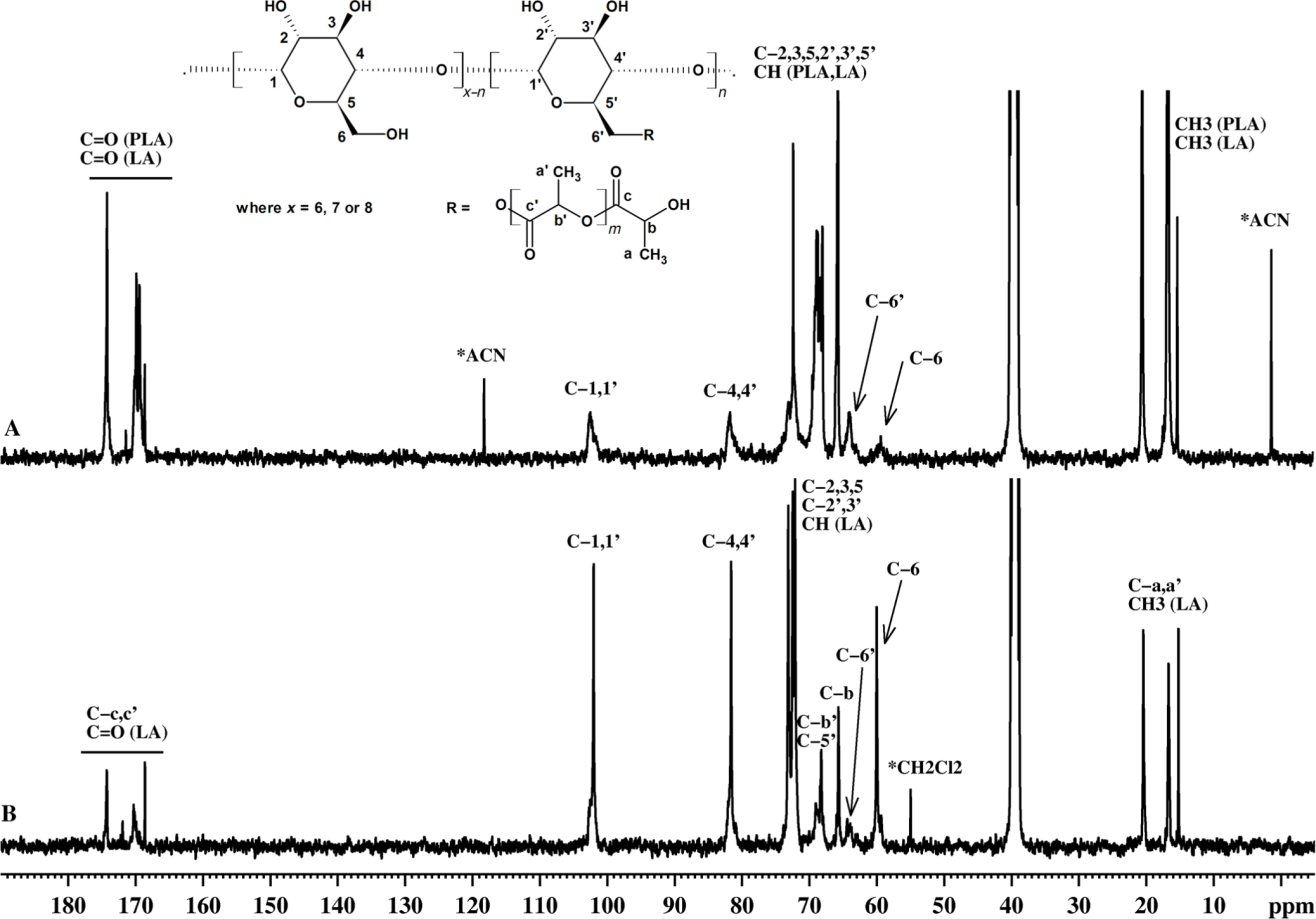
^13^C NMR spectra of (A) β-CD-LA F2 fraction and (B) β-CD-LA F1 fraction.

In the ^13^C NMR spectra of β-CD-LA F1 and F2 (Figure 5), the peaks assigned to C5’ (69.1 ppm) and C6’ (64.4 ppm) of the substituted glucopyranose unit are clearly isolated. The rest of the peaks for the carbons of the substituted unit appear as a small broadening of the peaks for the unsubstituted units.

**Figure 6:**
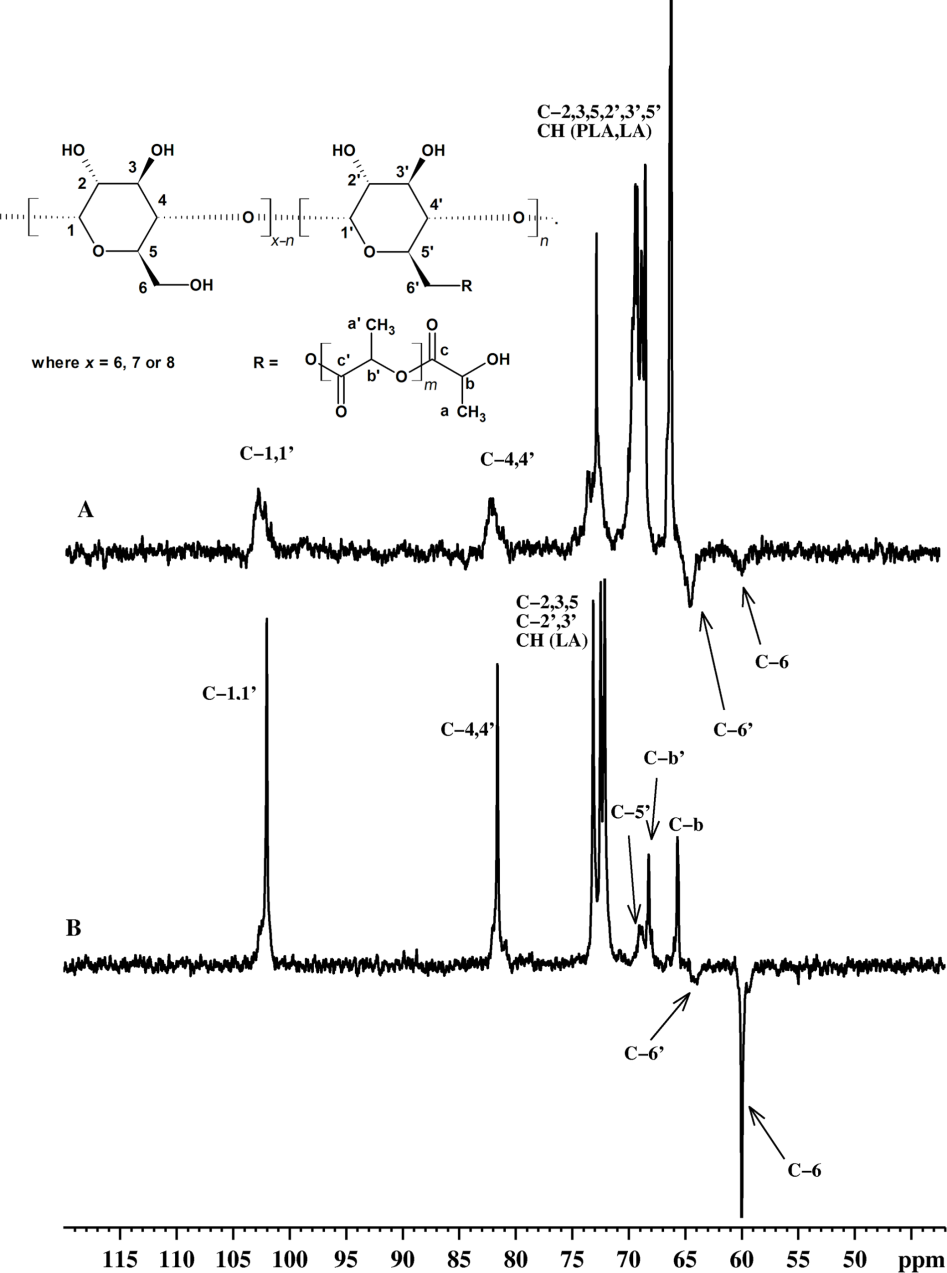
DEPT135-NMR experiment of (A) β-CD-LA F2 fraction and (B) β-CD-LA F1 fraction.

Similar compounds (CDs esterified with δ-valerolactone, β-butyrolactone and ε-caprolactone obtained by bulk polymerization) were reported by Harada et al. and were also analyzed by ^13^C NMR [17]. The structural assignment of the esterified CDs was supported in Harada’s work [17] by comparing the NMR spectra with those obtained for a monoesterified β-cyclodextrin at C2 position (mono-2-O-(6-benzyloxypentanoyl)-β-cyclodextrin). The peak at 63.4 ppm was assigned as C2’ of the monosubstituted glucose ring belonging to mono-2-*O*-(6-benzyloxypentanoyl)-β-cyclodextrin. The peak at 64.4 ppm, observed in our experiment, was differently assigned using a DEPT135 experiment on β-CD-LA-F1 and F2 (Figure 6). In the DEPT135-NMR spectra the peaks at 60 and 64.4 ppm are in opposite phase compared to the CH and CH_3_ peaks, indicating that they correspond to CH_2_ units; therefore, we assigned the peak observed in the same region for our compounds as esterified C6’. Interestingly, our structural assignment is in good agreement with the study published by Shen et al. [16] for solution ring opening of DL-LA initiated by β-CD and catalyzed by DMF. While, in their case the presence of DMF can justify an activation mechanism of the OH groups by the amines (similar with the mechanism proposed by Zinck et al. [6]), in the present work the activation mechanism of OH groups is not applicable.

By comparing the ^13^C NMR spectra of F1 and F2 fractions of β-CD-LA it may be observed that the intensity ratios of the C6 and C6’ are reversing with increase of the lactate moieties amounts per CD (Figure 5 and Figure 6). This suggests that the degree of substitution at the C6 is also increasing, thus being implied that for higher degrees of polymerization more glycoside rings are substituted predominantly at C6. This is also supported by the comparison between the ^1^H NMR integral ratios of OH2 and OH3 versus OH6 as previously discussed (Supporting Information File 1, Figure S27).

Therefore, we may conclude that the lactide ring opening is performed mostly by the OH groups from C6. The DEPT135-NMR experiment was also performed for α- and γ-CD-LA F1 samples (spectra given in Supporting Information File 1, Figures S28 and S29) confirming also the substitution at the C6.

The evaluation of the oligolactide chains attached to the CD may also include measuring of the average length. However, due to peak overlapping resulted from the presence of L-LA monomer and PLA homopolymer peaks this evaluation was not possible. However, the ratio between unsubstituted C6 and substituted C6’ corresponding peaks (Figure 5 and Figure 6) is changing, thus implying that cyclodextrins are substituted with more than one chain of oligolactide. If we take into consideration the β-CD-LA F2 sample, with an almost full substitution at C6 (approximately 6 glycosidic units of a total 7) and compare with the MALDI–MS results which gave an average of 36 lactate (18 dilactate) units per CD molecule we may infer that CDs can be tethered with 6 chains having an average length of 6 lactate units.

## Conclusion

Bulk polymerization of L-LA in the presence of α-, β- and γ-CD proceeds with the formation of oligolactides tethered cyclodextrins. In the conditions of monomer excess the initiator doesn’t fully react, free CDs being also detected in characterization by LC–ELSD with “off line” MALDI–MS detection. During this process PLA homopolymers are also formed, as confirmed by LC–ESI–MS analysis, possibly because of water traces in the reaction. The reactivity of different cyclodextrins in the ring opening of L-lactide may be summarized as follows: β-CD > α-CD > γ-CD. This statement is supported by liquid chromatography with evaporative light scattering detection providing the relative amount of free CD versus CD-LA products and PLA homopolymers. In addition, MALDI–MS characterization of the fractionated samples showed that the average molecular weight of β-CD-LA was the highest, with an average of 36 lactate units per cyclodextrin molecule. The NMR spectroscopy showed that the obtained products are best described as random-(6-*O*-oligolactide)cyclodextrins thus demonstrating a certain selectivity in the cyclodextrin’s modification. The presented combination of analytical methods can help in further studies to optimize the reaction conditions in order to achieve a modification of all CD molecules. Further characterization studies will aim for the quantitative measurement of cyclodextrin derivatives and PLA homopolymers in the reaction mixture.

## Experimental

### Materials

L-lactide (L-LA) (Sigma-Aldrich) was recrystallized twice from ethyl acetate, dried under vacuum and sublimated before use; α-, β- and γ-cyclodextrins (CDs) (Cyclolab, Hungary) were dried over P_2_O_5_ under vacuum at 80 °C for 72 hours and kept over P_2_O_5_ in the desiccator under Ar atmosphere. All used solvents were HPLC grade and were used as received.

### Instruments

The HPLC system consisted of a gradient pump of the Agilent 1260 series (Agilent Technologies, USA) coupled with an evaporative light scattering detector (ELSD) model ELS-1000 from PL-Agilent Technologies, Stretton, UK. Mass spectra of polymers were measured on an UltrafleXtreme TOF instrument (Bruker), equipped with a 355 nm smartbeam-2 laser, capable of a pulsing frequency of 1 kHz. The mass spectrometer was operated by FlexControl 3.3 software (Bruker). The acquired spectra were processed by FlexAnalysis 3.3 software (Bruker). LC–ESI–MS experiments were conducted using the AGILENT 6520 LC QTOF MS equipped with a dual ESI source. The data were analyzed using the Mass Hunter software. The NMR spectra were recorded on a Bruker Avance DRX 400 MHz Spectrometer equipped with a 5 mm QNP direct detection probe and z-gradients. Spectra were recorded in DMSO-*d*_6_, at room temperature. The chemical shifts are reported as δ values (ppm) and were referenced to the solvent residual peak (2.512 ppm for ^1^H and 39.47 ppm for ^13^C). The assignments of the peaks from the 1D NMR spectra were performed using 2D NMR experiments (H,H-COSY, H,C-HMQC, H,C-HMBC). The molecular parameters of the cyclodextrin–oligolactide covalent conjugates were also determined by GPC using a Shimadzu LC-20 isocratic pump and a Shimadzu refractive index detector in size exclusion mode using a PSS PFG precolumn and three PPS PFG columns (*d* = 8 mm, l = 300 mm) filled with particles with a size of 7 μm and pore sizes of 100, 300 and 1000 Å, respectively. 2,2,2-Trifluoroethanol was used as an eluent. Poly(methyl methacrylate) standards were used for the internal calibration.

### Methods

#### Bulk polymerization of L-LA

In a typical reaction 0.2 g of CD and 2.66 g of L-LA were weighted together under protection of Ar flow, in a dried flask containing a magnetic stirrer. The molar ratio between the L-LA and OH groups of CD was kept at a 5:1 value for all the polymerizations. All operations were conducted carefully under Ar atmosphere. The flask isolated with a rubber septum was completely immersed in an oil bath, over a heater with magnetic stirring and the temperature was brought to 110 °C. The L-LA monomer was quickly melted and the CDs were homogenously dispersed in the reaction mixture. The heating was maintained for 72 h under continuous stirring. The reaction was stopped by simply removing the flask from the heating source. In order to analyze the products, the samples were fractionated by three times washing with THF. Thus, two fractions were obtained for each reaction system, a fraction precipitated in THF, rich in free CDs or CD molecules with low substitution degree, noted with F1, and a second fraction, fully soluble in THF (composed of three fractions resulted from combining the resulted solutions from three repeated washing procedures), containing CDs with high substitution degree and unreacted L-LA monomer, noted with F2. The F2 fraction was further purified by partial removal of unreacted monomer through sublimation under vacuum at 40 °C temperature, in order to facilitate the NMR characterization. The unfractionated samples were first assessed by liquid chromatography with evaporative light scattering detection (LC–ESLD) "on line" and also using "off line" MALDI–MS. The F1 and F2 fractions obtained for each type of CD (α-CD-LA F1 – 2%) and F2 (98%), β-CD-LA F1 (5.5%) and F2 (94.5%) and γ-CD-LA F1 (9%) and F2 (91%) were characterized by MALDI–MS, NMR spectroscopy while the F2 fraction was also characterized by gel permeation chromatography (Table 1).

**α-CD-LA F1**: ^1^H NMR (400.13 MHz, DMSO-*d*_6_, δ ppm) 5.60–5.45 (OH2, OH3, end chain OH), 5.11 (CH-b’), 4.81 (H1), 4.51 (OH6), 4.29–4.19 (H6’, CH-b), 3.94 (H5’), 3.78 (H5), 3.68–3.58 (H3, H6), 3.40 (H4), 3.29 (H2), 1.47–1.43 (CH3-a’), 1.31–1.29 (CH3-a); ^13^C NMR (100.6 MHz, DMSO-*d*_6_, δ ppm) 174.01 (C-c), 170.11 (C-c’), 101.7 (C1), 81.8 (C4), 73.0 (C3), 71.8 (C2, C5), 68.2 (C5’), 67.9 (C-b’), 65.3 (C-b), 64.2 (C6’), 59.7 (C6), 20.1 (C-a), 16.5 (C-a’).

**β-CD-LA F1**: ^1^H NMR (400.13 MHz, DMSO-*d*_6_, δ ppm) 5.92–5.69 (OH2, OH3), 5.47–5.42 (end chain OH, CH from L-LA), 5.10 (CH-b’), 4.84 (H1), 4.47 (OH6), 4.31–4.19 (H6’, CH-b), 3.86 (H5’), 3.64–3.56 (H3, H5, H6), 3.38–3.10 (H2 and H4 overlapped with water from solvent), 1.47–1.42 (CH3 from L-LA, CH3-a’), 1.3–1.29 (CH3-a); ^13^C NMR (100.6 MHz, DMSO-*d*_6_, δ ppm) 174.2 (C-c), 170.3 (C-c’), 170.3 (C=O from L-LA), 102.0 (C1), 81.6 (C4), 73.1 (C3), 72.5 (C2), 72.1 (C5), 69.1 (C5’), 68.2 (C-b’), 65.7 (C-b), 64.4 (C6’), 60.0 (C6), 20.4 (C-a), 16.7 (C-a’), 15.2 (CH3 from L-LA).

**γ-CD-LA F1**: ^1^H NMR (400.13 MHz, DMSO-*d*_6_, δ ppm) 5.96–5.75 (OH2, OH3), 5.49–5.43 (end chain OH, CH from L-LA), 5.10 (CH-b’), 4.90 (H1), 4.55 (OH6), 4.28–4.2 (H6’, CH-b), 3.83 (H5’), 3.63–3.53 (H3, H5, H6), 3.83–3.3 (H2 and H4 overlapped with water from solvent), 1.47–1.40 (CH3 from lactide, CH3-a’), 1.31–1.29 (end chain CH3-a); ^13^C NMR (100.6 MHz, DMSO-*d*_6_, δ ppm) 174.2 (C-c), 170.2 (C-c’), 101.7 (C1), 81.0 (C4), 73.0 (C3), 72.6 (C2), 72.2 (C5, CH from L-LA), 68.8 (C5’), 68.2 (C-b’), 65.6 (C-b), 64.2 (C6’), 60.0 (C6), 20.4, 16.7 (CH3), 15.2 (CH3 from lactide).

**β-CD-LA F2**: ^1^H NMR (400.13 MHz, DMSO-*d*_6_, δ ppm) 5.94–5.70 (OH2, OH3), 5.5–5.42 (end chain OH, CH from L-LA), 5.20–5.12 (CH), 4.85 (H1), 4.65–4.18 (OH6, H6’, CH-b), 3.90 (H5’), 3.64–3.37 (H3, H5, H6, H2, H4), 1.49–1.41 (CH3 from L-LA, CH3), 1.3–1.28 (CH3); ^13^C NMR (100.6 MHz, DMSO-*d*_6_, δ ppm) 174.2–168.6 (C=O), 102.4 (C1, C1’), 81.7 (C4, C4’), 73.0 (C3, C3’), 72.2–65.7 (C2, C2’, C3, C3’, C5, C5’, CH), 63.9 (C6’), 59.3 (C6), 20.4–16.59 (CH3).

The peaks from the ^1^H NMR spectra for the F2 fractions of α- and γ-CD-LA samples cannot be exactly assigned due to the large amount of free PLA homopolymer. The CH and OH protons from the PLA homopolymer give peaks in the 5.5–4 ppm region and they overlap with those from α- and γ-CD-LA so their separate integration is not possible. The ^13^C NMR spectrum for the α-CD-LA-F2 sample shows peaks for C6 at 59.55 ppm, for C6’ at 64.05 ppm, for C1 and C1’ at 102.2–101.6 ppm and for C4 and C4’ at 81.9 ppm. The ^13^C NMR spectrum for the γ-CD-LA-F2 shows peaks for C6 at 60.03 ppm, for C6’ at 63.59 ppm, C1 and C1’ at 102–101 ppm and for C4, C4’ at 80.9 ppm. The rest of the peaks from the ^13^C NMR spectra for F2 fractions of α- and γ-CD-LA samples cannot be fully assigned due to the large amount of free PLA homopolymer: 174–168 ppm (-CO), 72–65 ppm (-CH-), 20–15 ppm (-CH3).

#### Liquid chromatography with evaporative light scattering detection (LC ELSD)

Temperature of evaporator was set at 80 °C and gas flow rate was 1.5 mL·min^−1^. HPLC column was Purospher Star RP-18 end capped column (5 µm) 250 × 4.6 mm purchased from Merck, Germany. The mobile phase consisted of (A) water and (B) acetonitrile. Gradient elution was realized as follows: solvent (A) was maintained at 90% for 3 min, followed by linear gradient to 10% of (A) in 10 min. These conditions were held for 5 min. The initial conditions were obtained in 5 min and to recondition the column 3 min post-run with the initial mobile phase composition was performed. Injecting volume was 20 μL. The flow rate of mobile phase was set on 1 mL/min. The HPLC measurements were performed at ambient temperature. Data were collected and processed using the software Clarity from DataApex, Czech Republic.

#### Matrix-assisted laser desorption ionization (MALDI–MS)

The raw samples withdrawn directly from the polymerization mixture were dissolved in a 1:1 water/acetonitrile mixture to a concentration of 10 mg/mL. The liquid chromatography fractions were used as such. Samples were mixed with a matrix solution (saturated solution of α-cyano-hydroxy-cinnamic acid in water/acetonitrile mixture) in a ratio of 1:100 (v/v). 1 μL of this mixture was deposited on polished steel MALDI target (Bruker). The ionization laser power was adjusted just above the threshold in order to produce charged species. The mass spectra were collected in amount of above 10000 spectra for each sample.

#### Liquid chromatography with electrospray ionization mass spectrometry detection (LC–ESI–MS)

The ESI–MS parameters were set as follows: Vcap = 4000 V, fragmentor voltage = 200 V, drying gas temperature = 325 °C, drying gas flow = 10 L/min and nebulizer pressure = 35 psig. Nitrogen was used as spraying gas. LC separations were performed by using a C18 column - Agilent ZORBAX 300SB-C18 4.6 × 150 mm, 5 μm. The samples were separated by gradient elution using water/acetonitrile solvent mixture at 26 °C constant temperature in column compartment. The used eluents were: A – 2 mM formic acid solution and B – acetonitrile. The samples were solved in a 1:1 (vol/vol) water/acetonitrile mixture and 10 μL were injected. For the LC–MS analysis of β-CD-LA, the mobile phase was delivered at 1 mL/min in linear gradient mode: 0–3 min, 20% B; 10 min, 100% B; 5 min, 100% B; 3 min, 20% B.

## Supporting Information

File 1 Analytical data.
