# Supplementary material for: Cyclodextrins tethered with oligolactides – green synthesis and structural assessment
Source: Beilstein J Org Chem. 2017 Apr 26;13:779–92. doi: 10.3762/bjoc.13.77 (PMC5433207; doi:10.3762/bjoc.13.77)
Supplement: File 1 — Analytical data. [file Beilstein_J_Org_Chem-13-779-s001.pdf]

Supporting Information

for

Cyclodextrins tethered with oligolactides – green synthesis and structural assessment

Cristian Peptu*[1,2], Mihaela Balan-Porcarasu[2], Alena Šišková[1], Ľudovít Škultéty[3] and Jaroslav Mosnáček[1]

Address: [1]Polymer Institute of Slovak Academy of Sciences, Dúbravská cesta 9, 84541 Bratislava, Slovakia, [2]"Petru Poni" Institute of Macromolecular Chemistry, Alee Grigore Gica Voda 41A, 700487 Iasi, Romania and [3]Institute of Virology, Biomedical Research Center Slovak Academy of Sciences, Dúbravská cesta 9, 84541 Bratislava, Slovakia

Email: Cristian Peptu - cristian_peptu@yahoo.com

*Corresponding author

Analytical Data

Contents

Liquid chromatography with detection by evaporative light scattering (LC ELSD) and matrix assisted laser desorption ionization mass spectrometry (MALDI–MS) Figures S1–S21 –pages S2–S12

LC with electrospray mass spectrometry (ESI–MS) detection of β-CD-LA S22, S23 – page S13

NMR spectra of CD-LA Figures S24–S44 –pages S14–S34

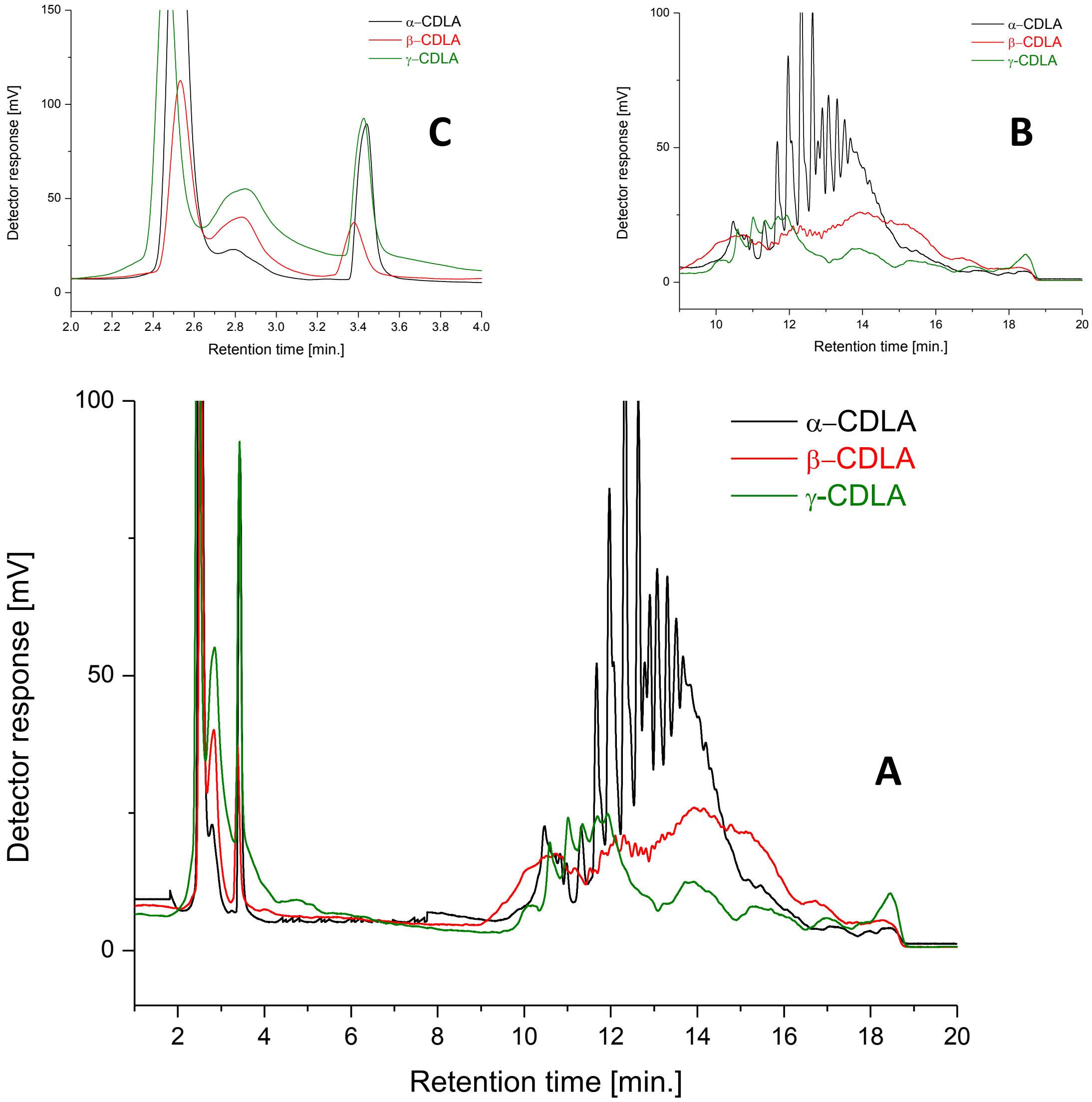

Figure S1: LC–ESLD chromatograms of α-, β- and γ-CD-LA (A), highlights of 9–20 min region (B) and 2–4 min region (C).

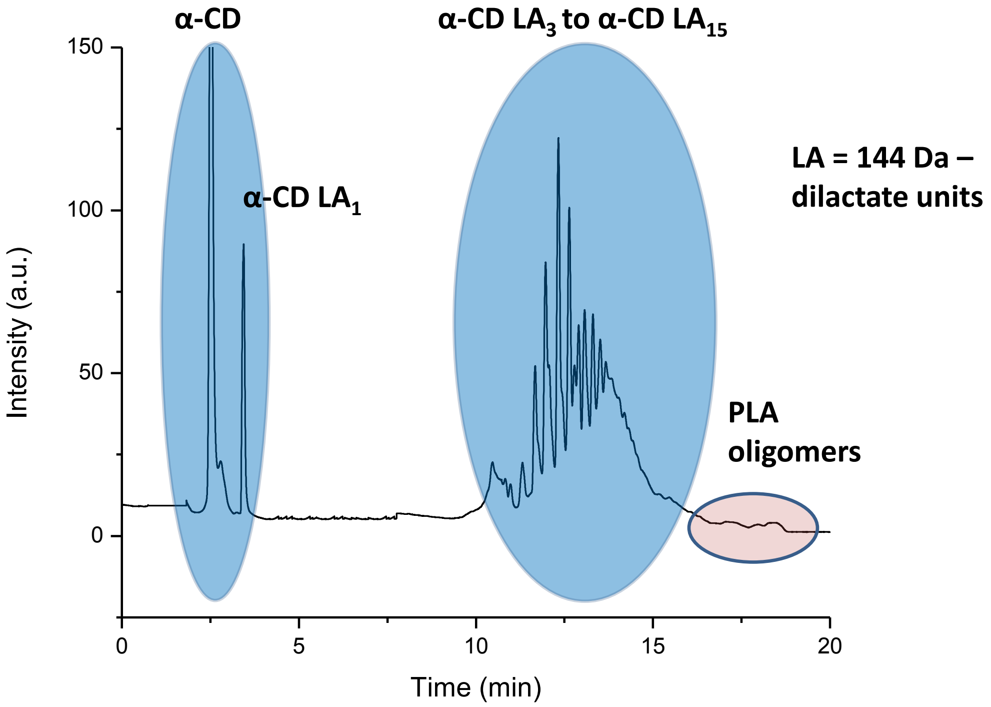

Figure S2: LC–ESLD chromatogram of α-CD-LA(f1 - 2.3 – 3 min; f2 – 3 – 4.2 min; f3 – 9.8 – 12 min; f4 – 12 – 14 min; f5 – 14 – 16 min; f6 – 16 – 19 min).

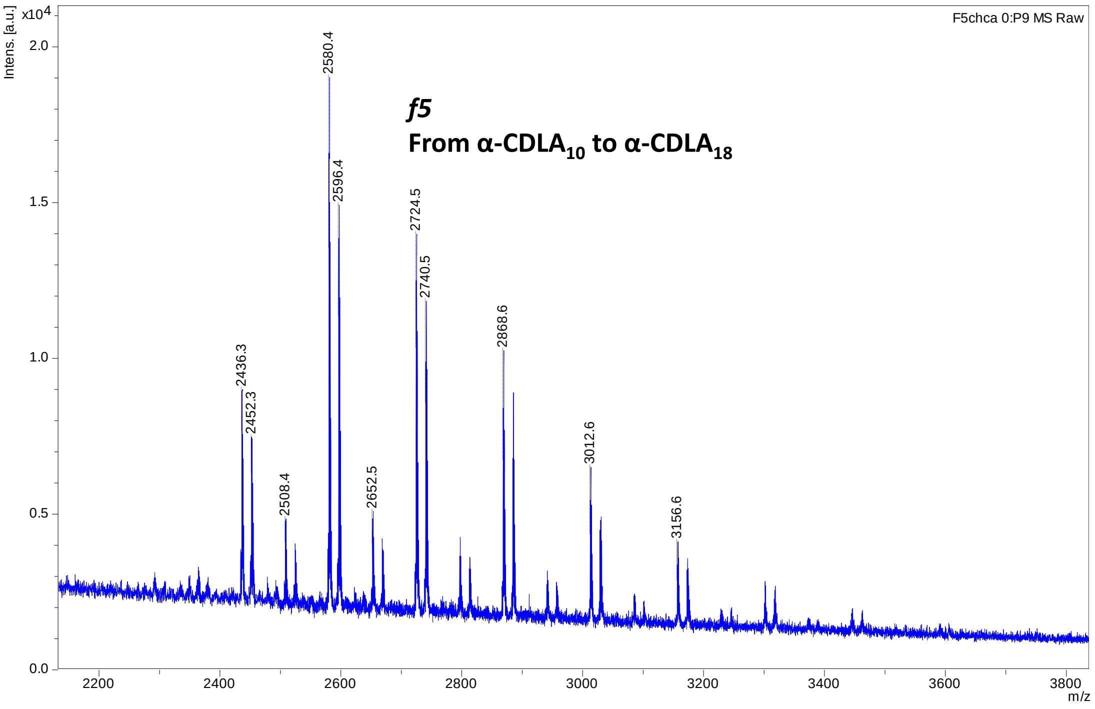

Figure S3: MALDI–MS spectrum of fraction f5 from α-CD-LA separation.

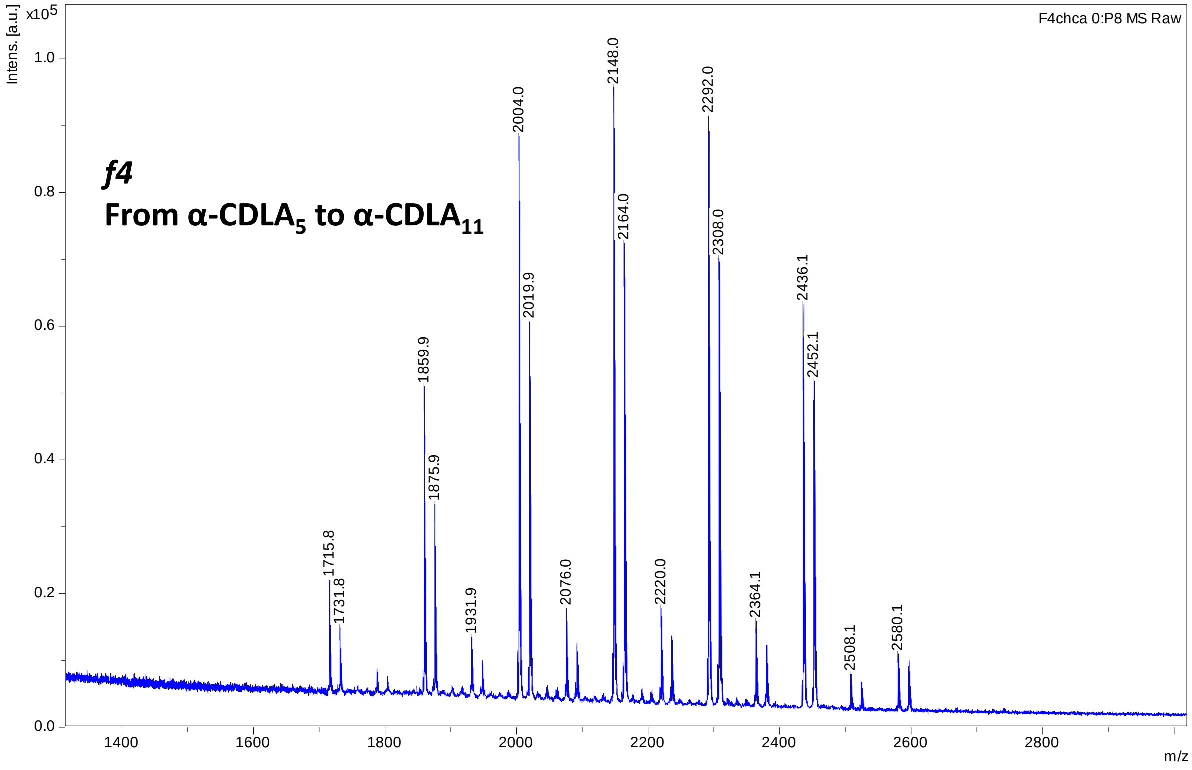

Figure S4: MALDI–MS spectrum of fraction f4 from α-CD-LA separation.

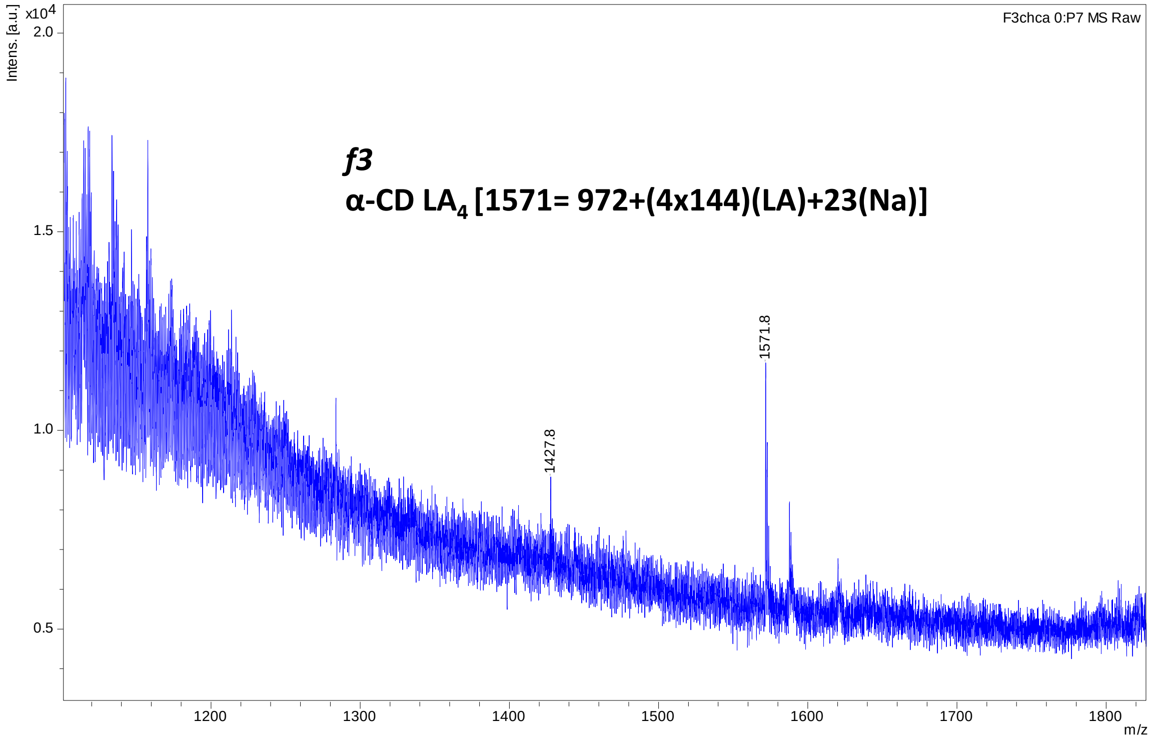

Figure S5: MALDI–MS spectrum of fraction f3 from α-CD-LA separation.

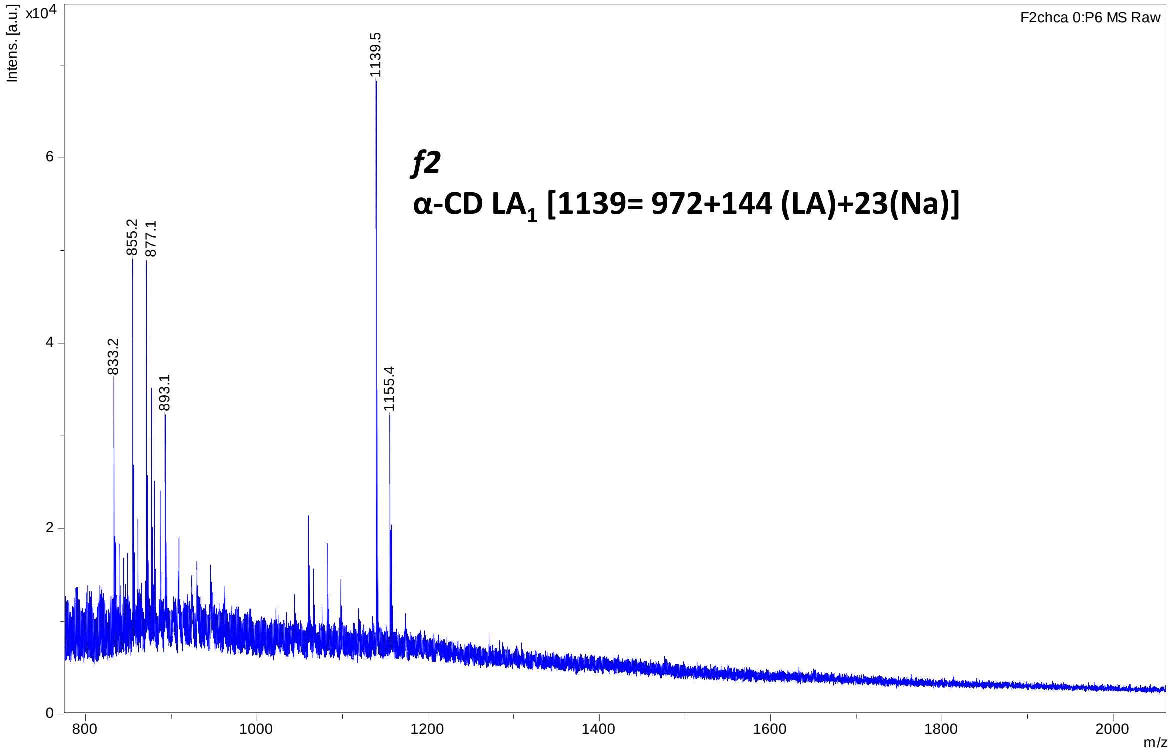

Figure S6: MALDI–MS spectrum of fraction f2 from α-CD-LA separation.

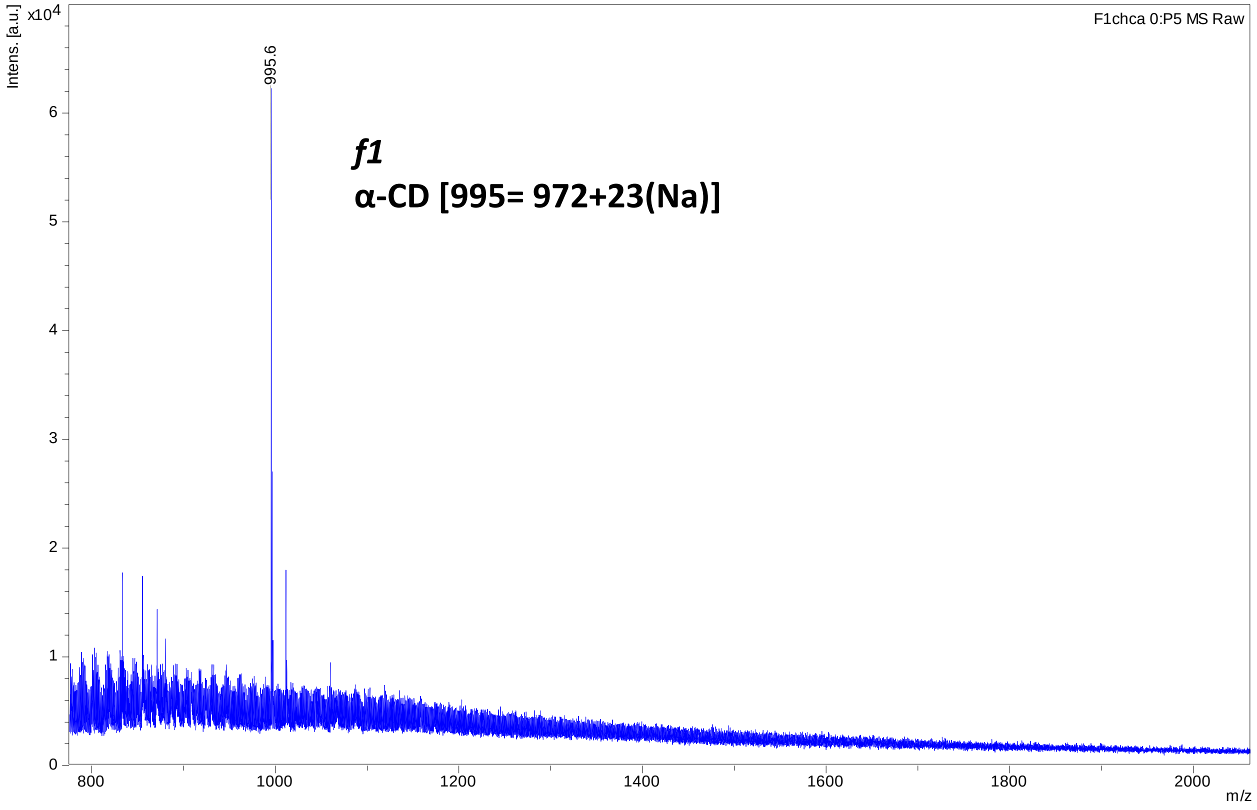

Figure S7: MALDI–MS spectrum of fraction f1 from α-CD-LA separation.

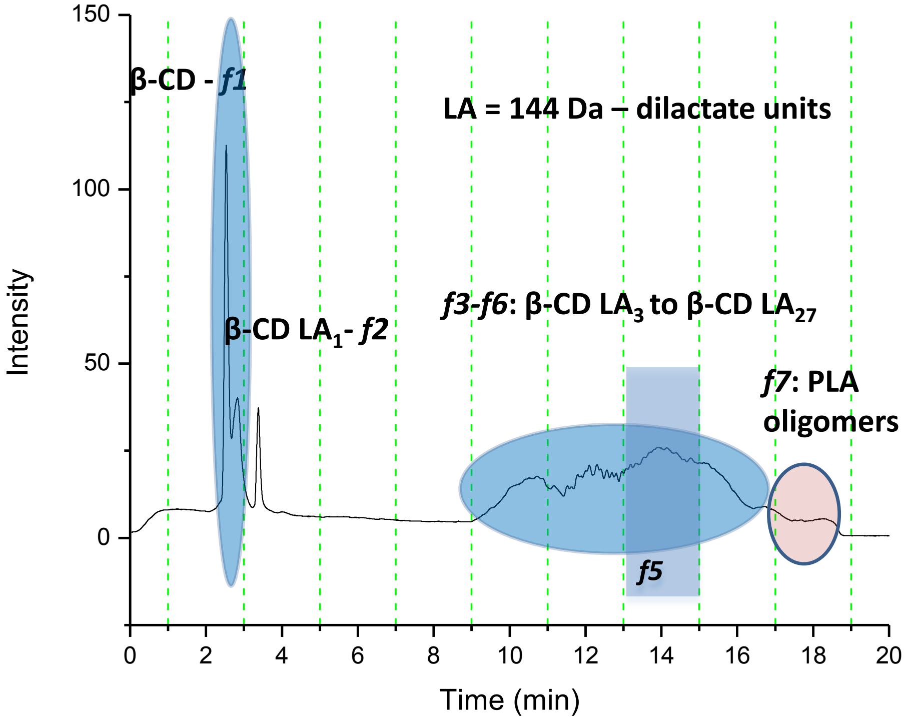

Figure S8: LC–ESLD chromatogram of β-CD-LA (f1 - 2– 3 min; f2 – 3 – 4 min; f3 – 9 – 11 min; f4 – 11 – 13 min; f5 – 13 – 15 min; f6 – 15 – 17 min; f7 – 17 – 19 min).

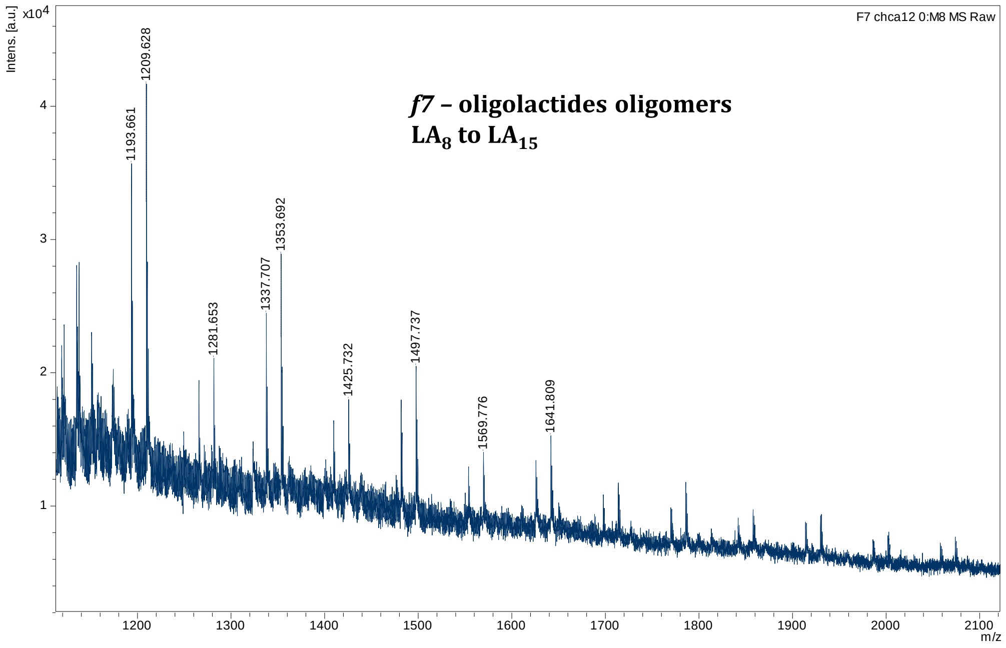

Figure S9: MALDI–MS spectrum of fraction *f7* from β-CD-LA separation.

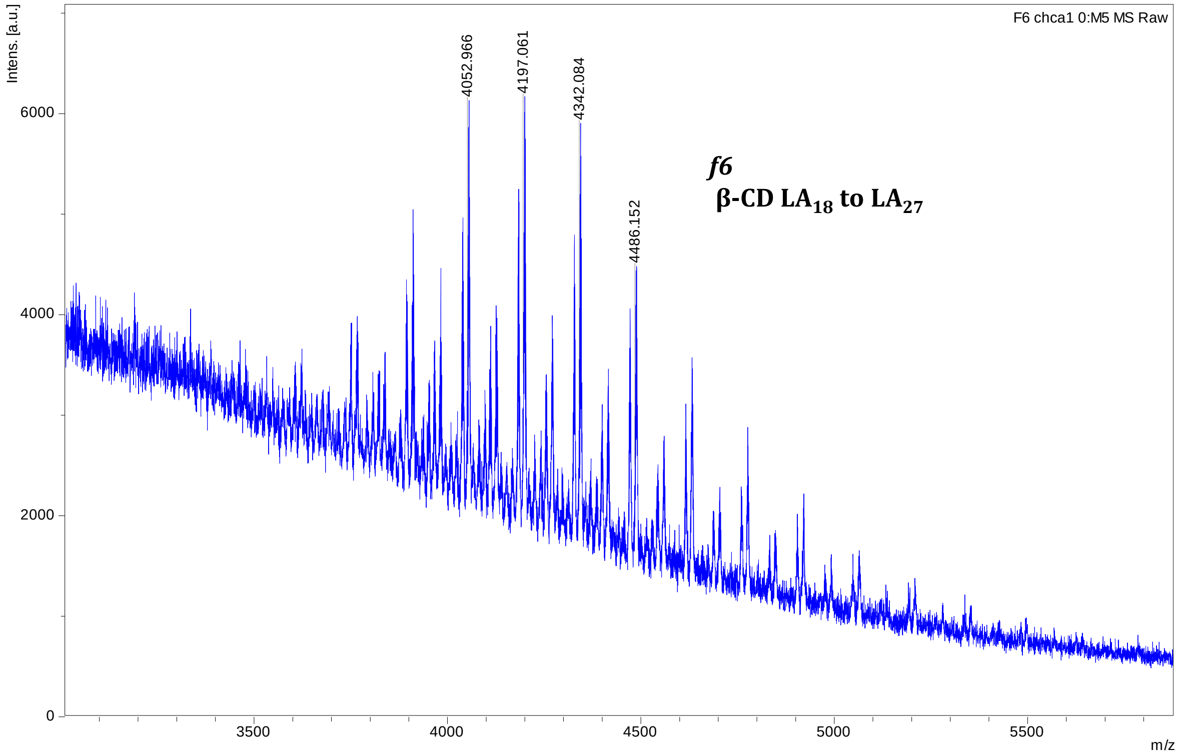

Figure S10: MALDI–MS spectrum of fraction *f6* from β-CD-LA separation.

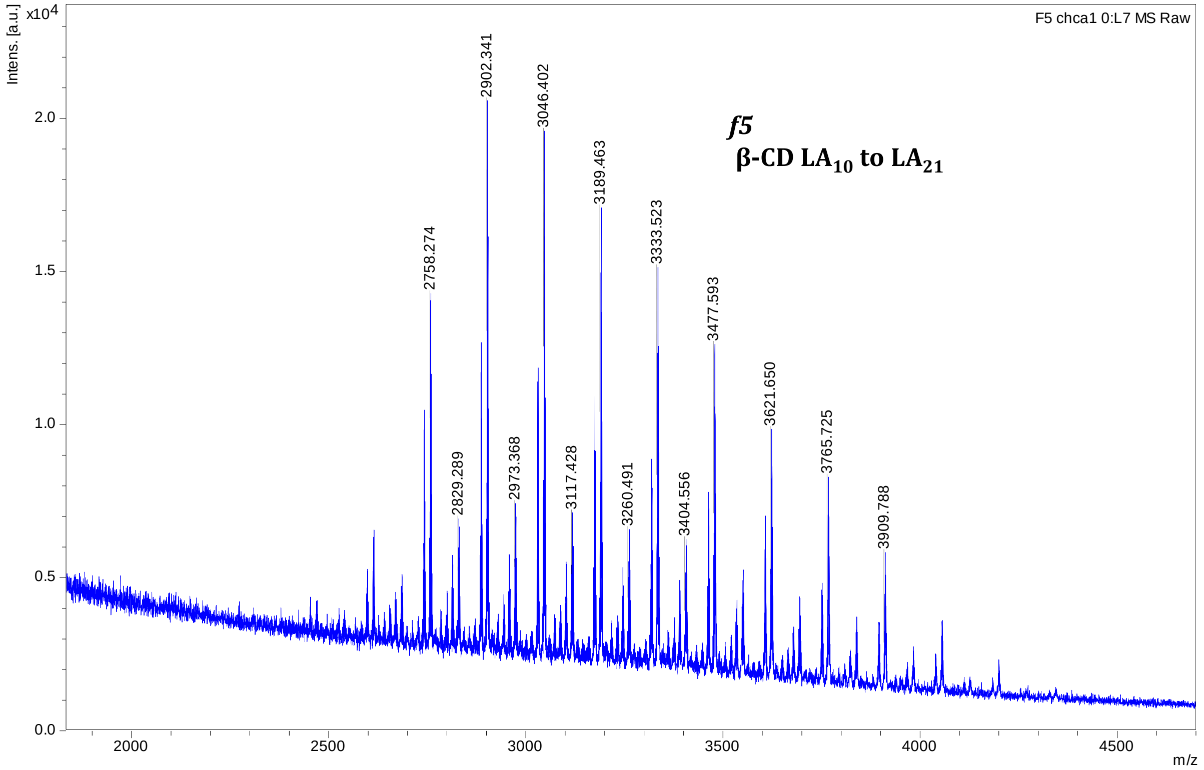

Figure S11: MALDI–MS spectrum of fraction *f5* from β-CD-LA separation.

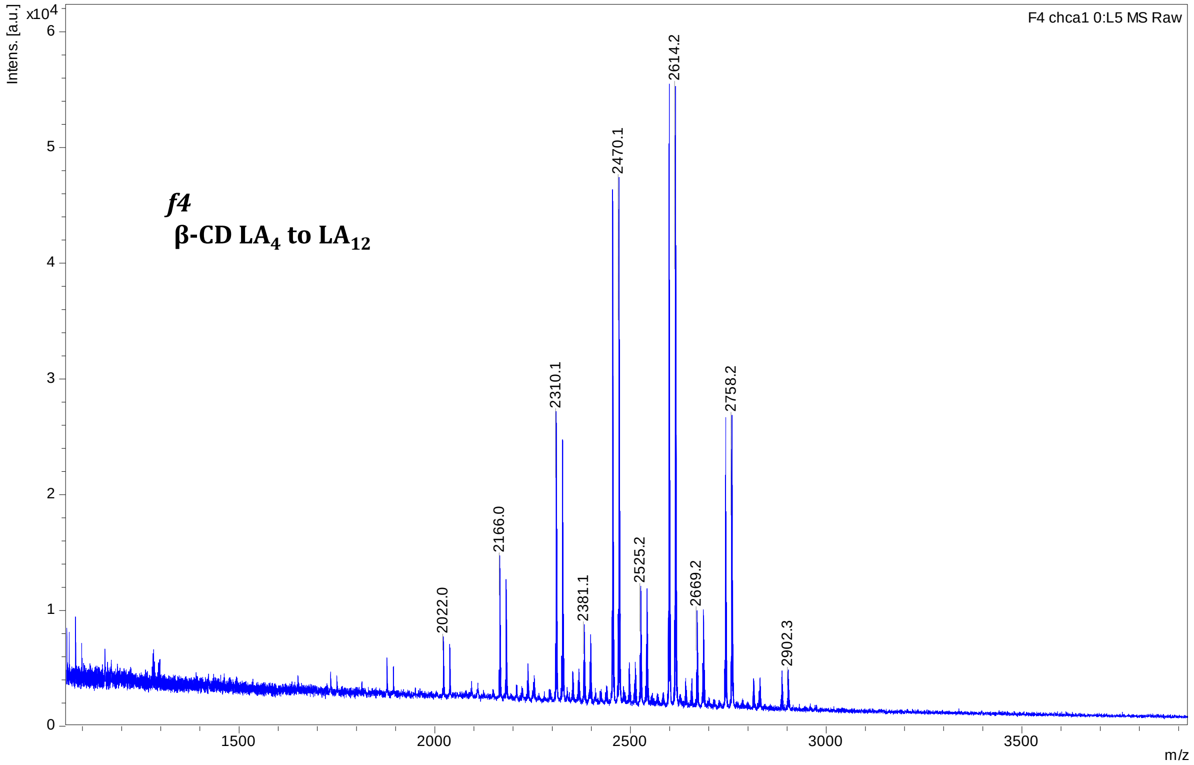

Figure S12: MALDI–MS spectrum of fraction *f4* from β-CD-LA separation.

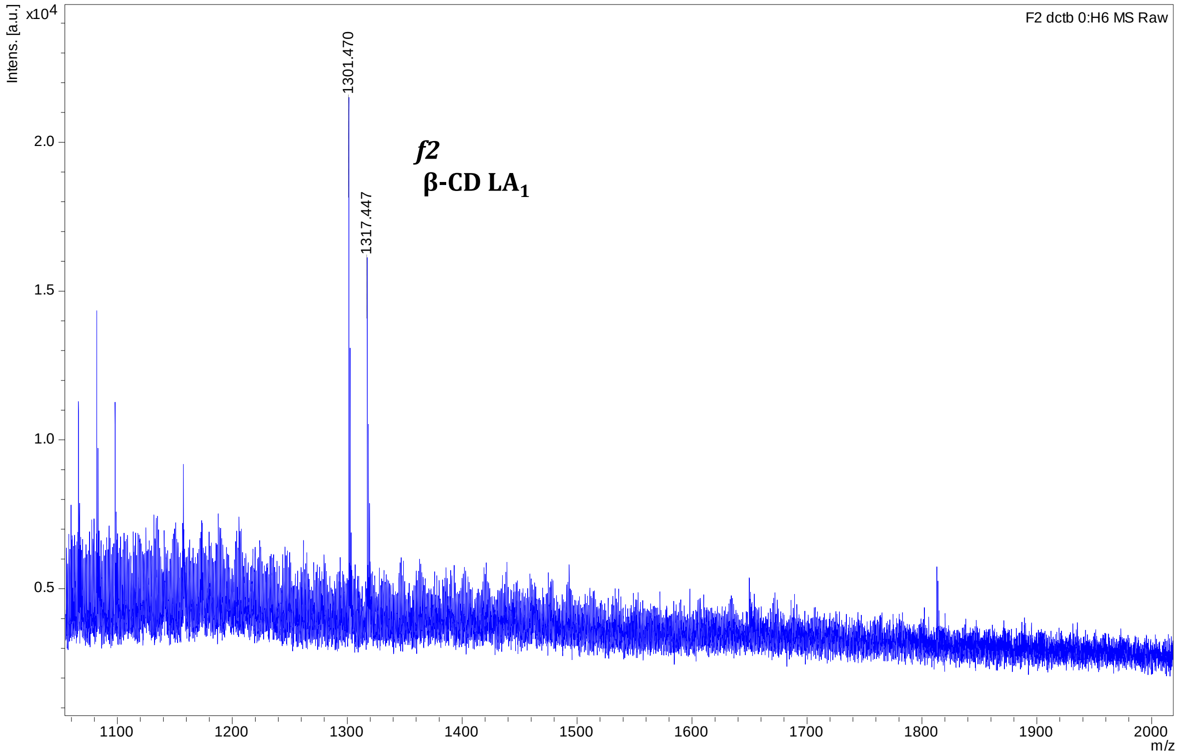

Figure S13: MALDI–MS spectrum of fraction *f2* from β-CD-LA separation.

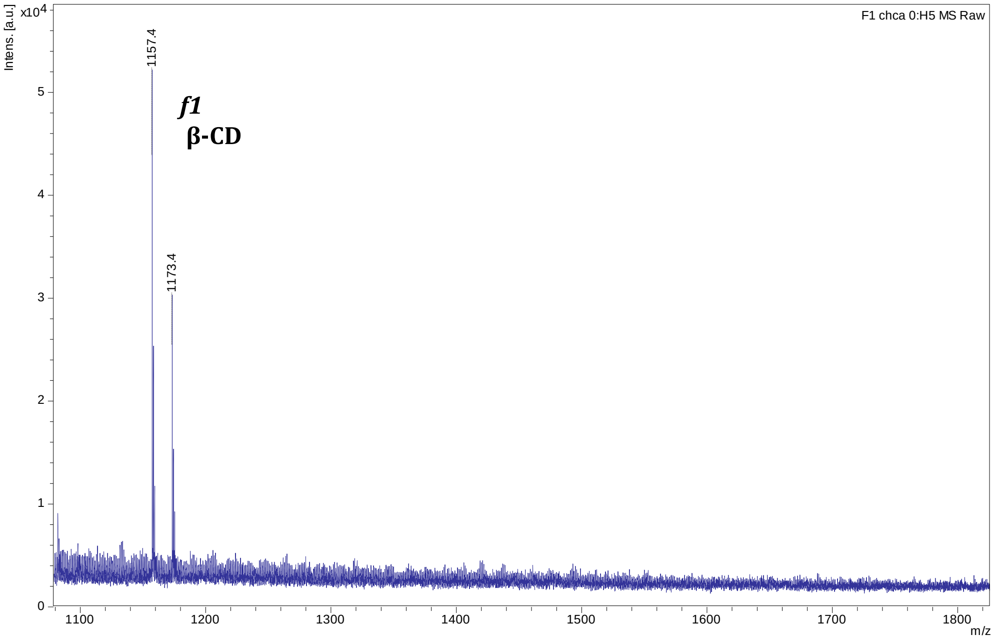

Figure S14: MALDI–MS spectrum of fraction *f1* from β-CD-LA separation.

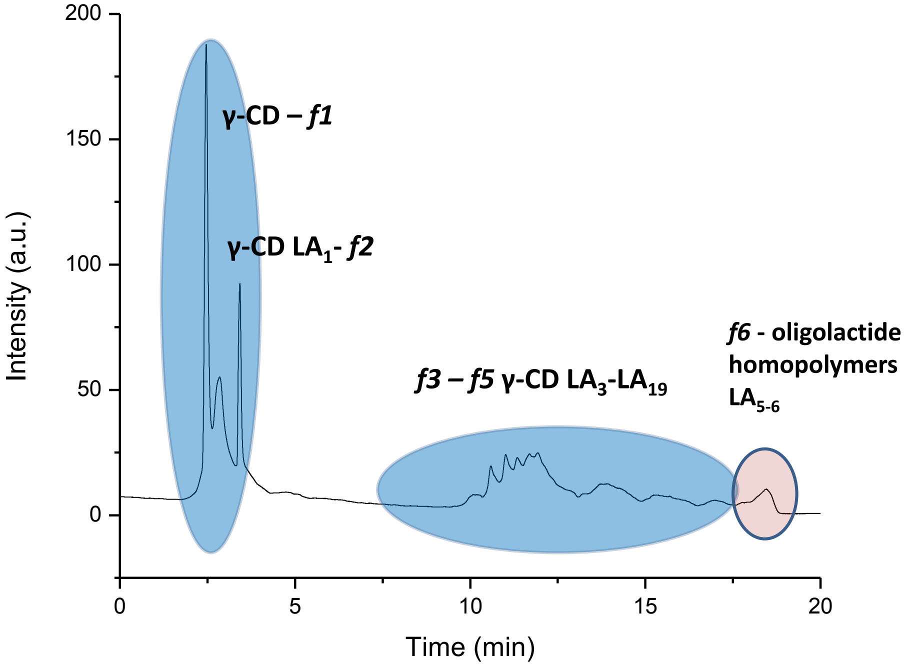

Figure S15: LC–ESLD chromatogram of γ-CD-LA (f1 - 2.3 – 3 min; f2 – 3 – 4.2 min; f3 – 10 – 12.5 min; f4 – 12.5 – 15 min; f5 – 15 – 17.5 min; f6 – 17.5 – 19 min).

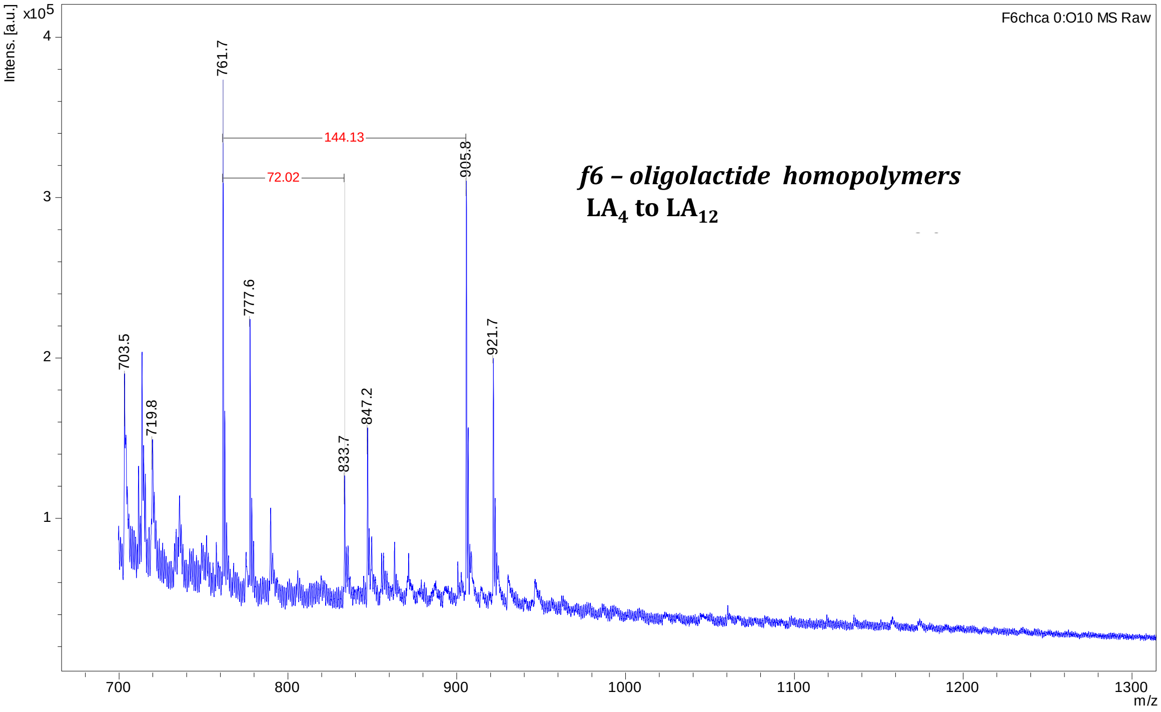

Figure S16: MALDI–MS spectrum of fraction *f6* from γ-CD-LA separation.

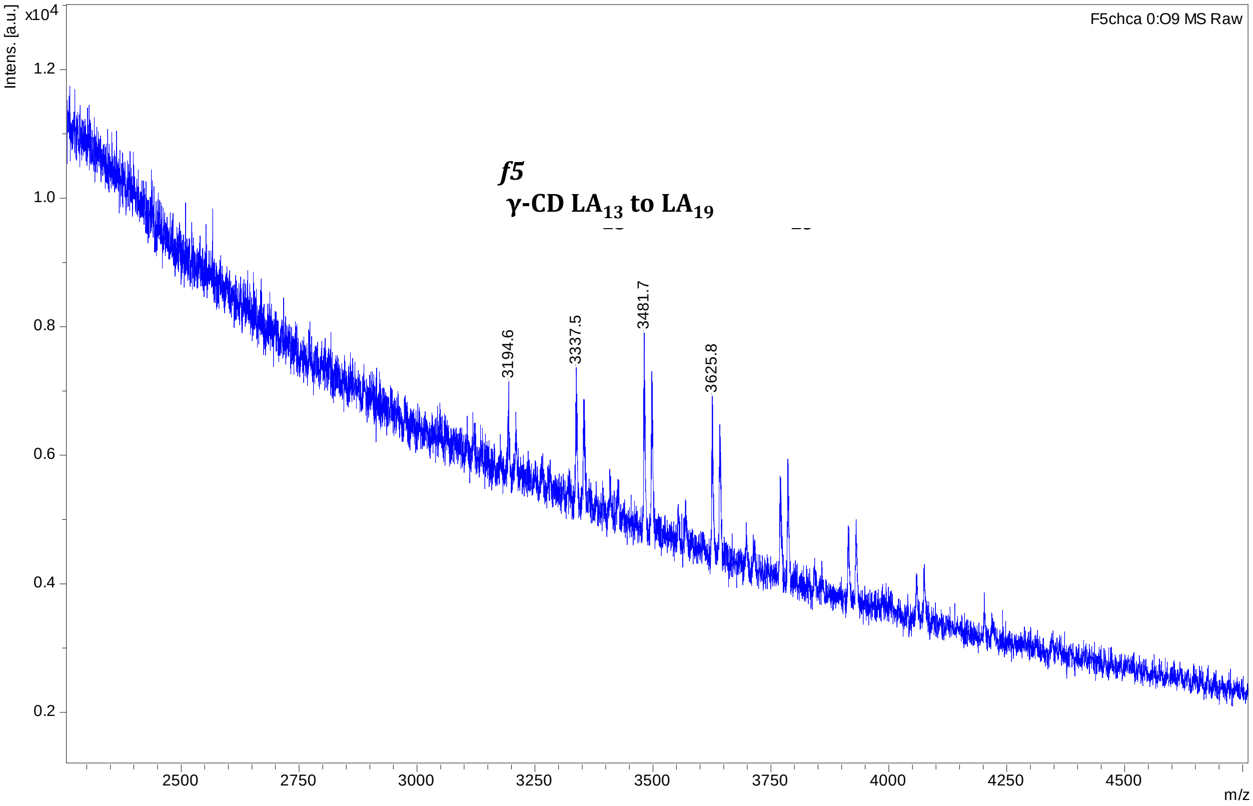

Figure S17: MALDI–MS spectrum of fraction *f5* from γ-CD-LA separation.

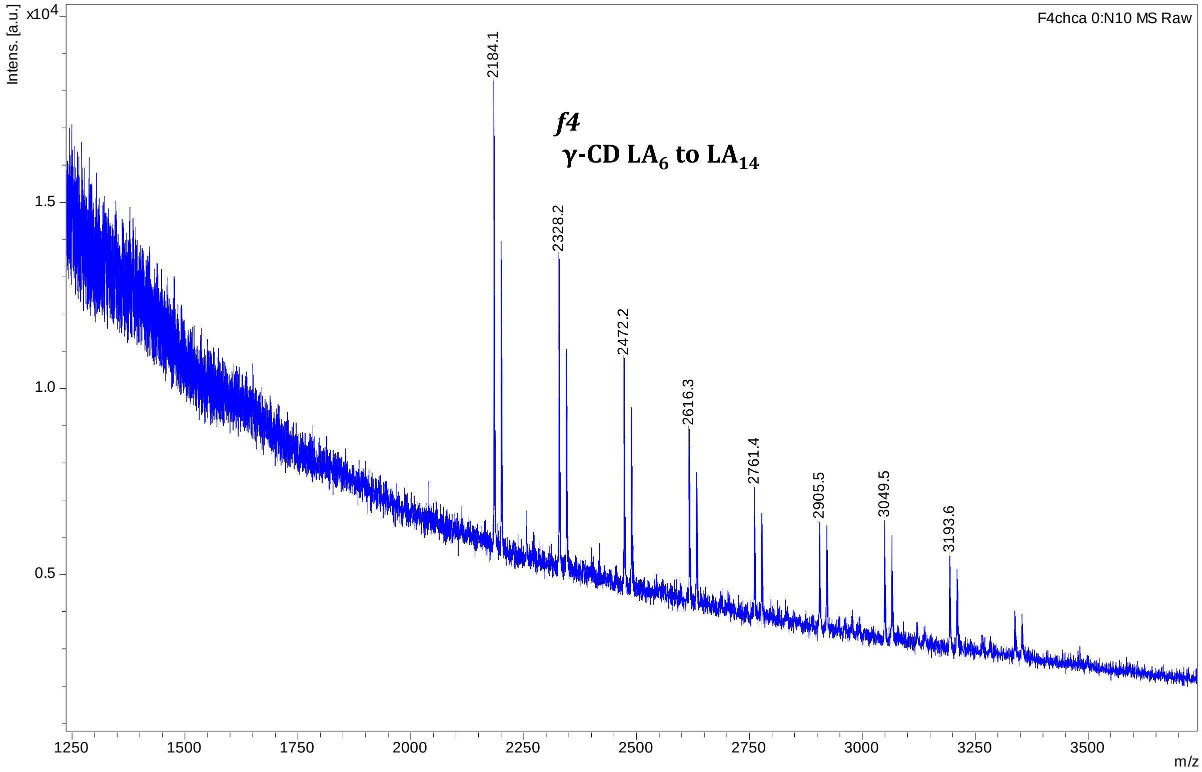

Figure S18: MALDI–MS spectrum of fraction *f4* from γ-CD-LA separation.

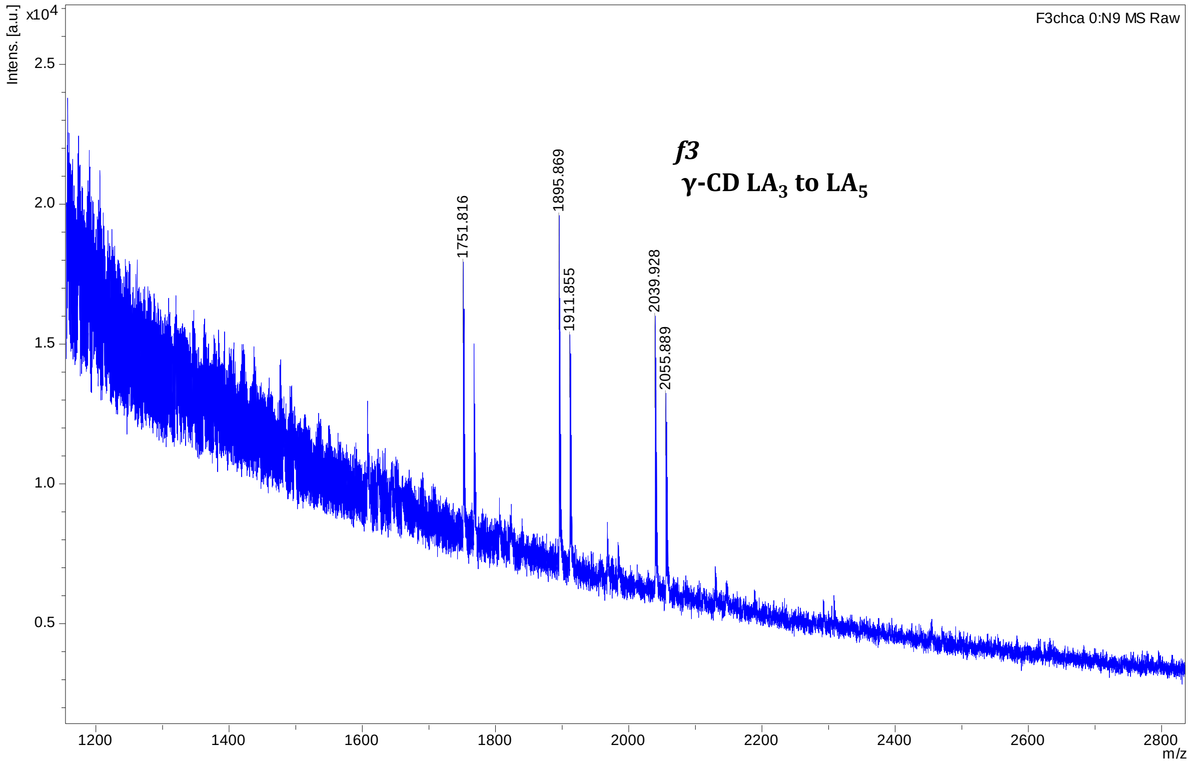

Figure S19: MALDI–MS spectrum of fraction *f3* from γ-CD-LA separation.

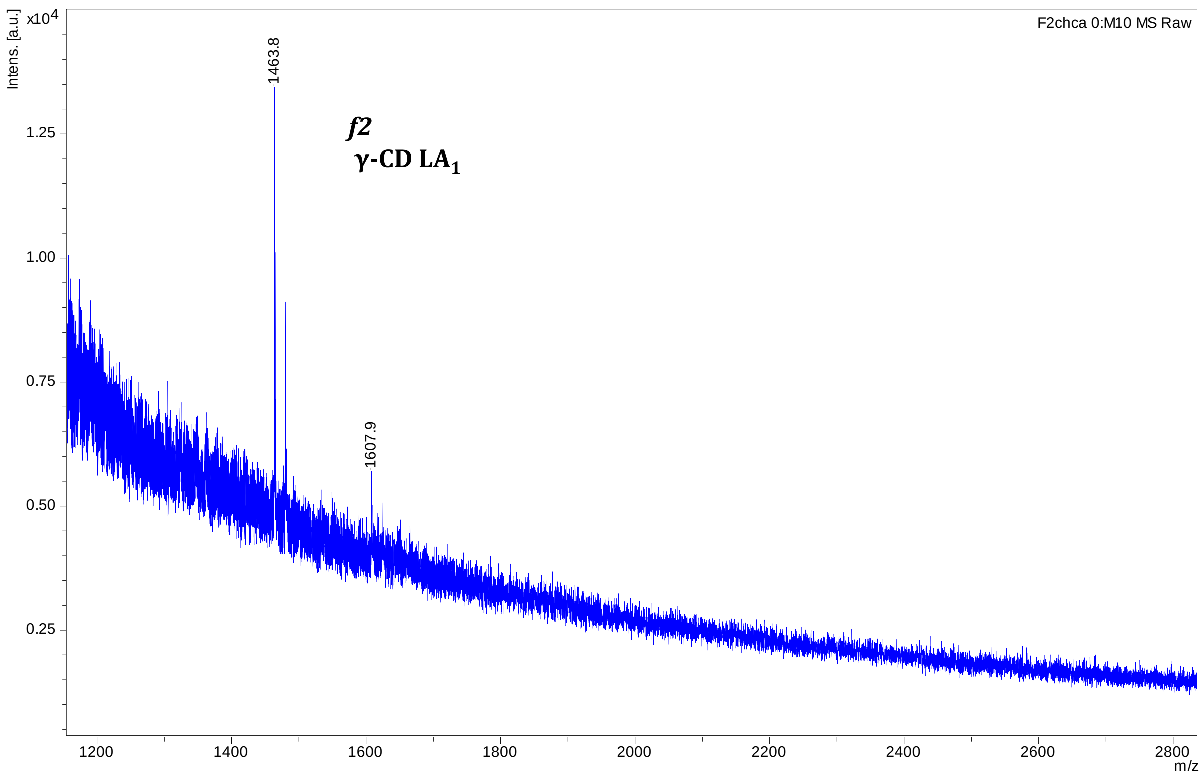

Figure S20: MALDI–MS spectrum of fraction *f2* from γ-CD-LA separation.

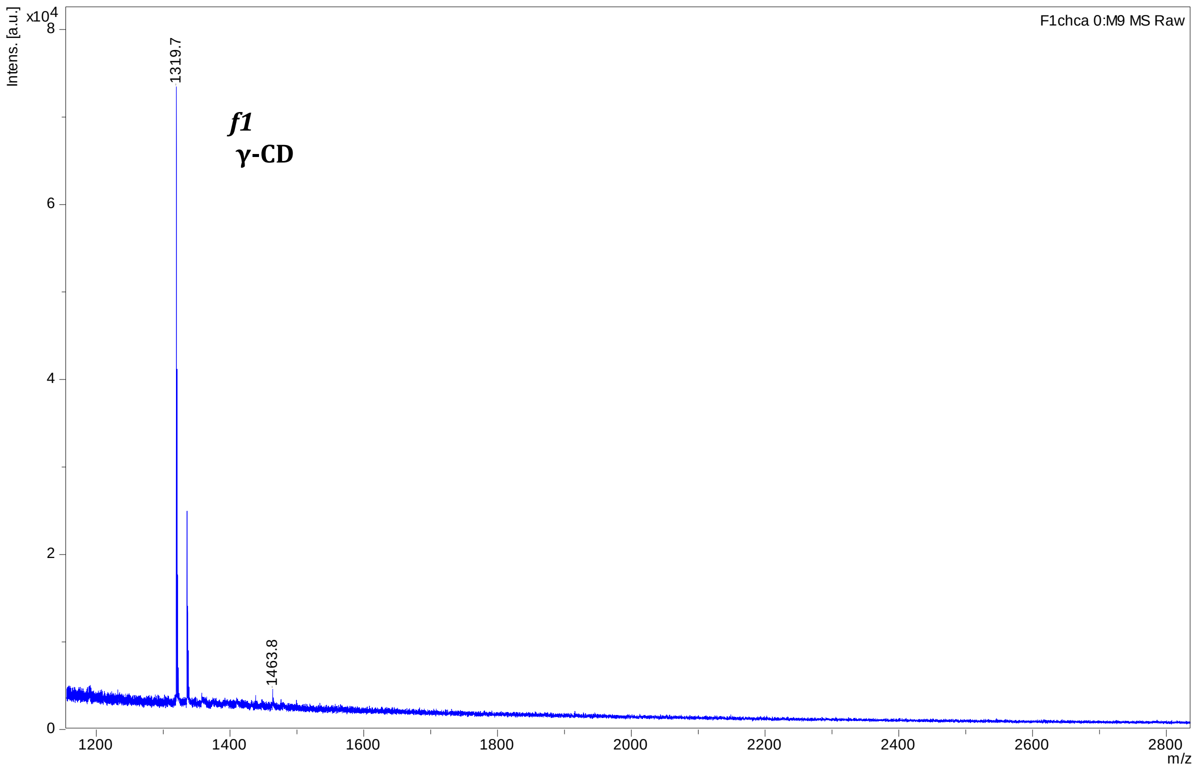

Figure S21: MALDI–MS spectrum of fraction *f1* from γ-CD-LA separation.

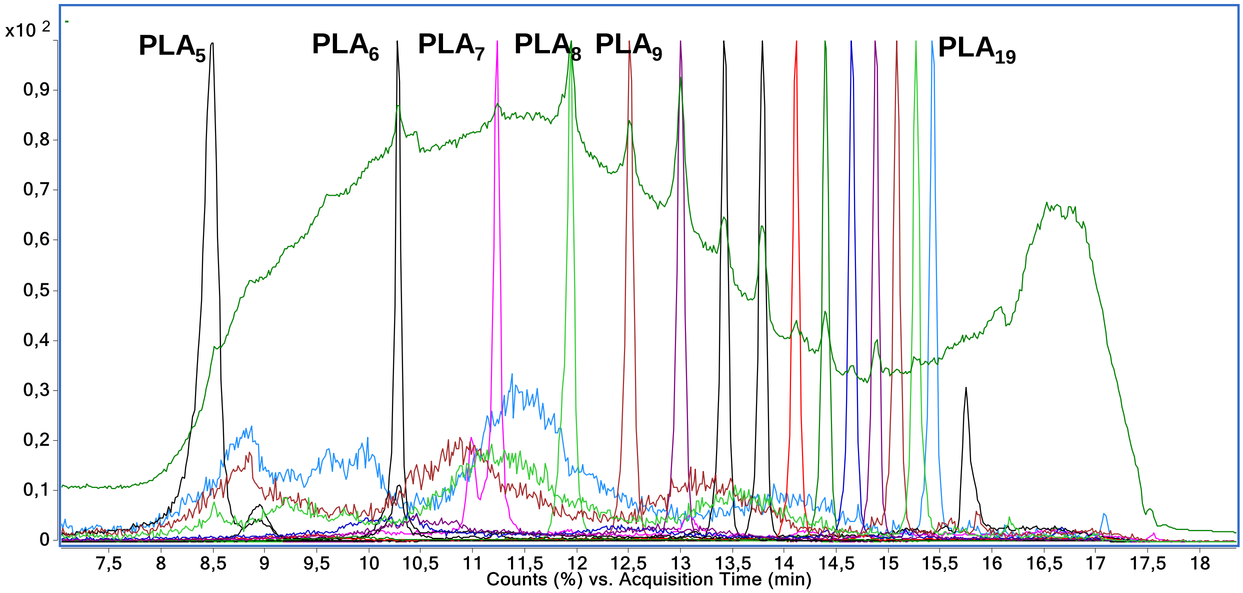

Figure S22: LC–MS separation of β-CD-LA (total ion chromatogram from 8 to 17.5 min and extracted ion chromatograms of PLA oligomers from 5 to 19 lactate units).

In Figure S22 are overlapped the extracted ion chromatograms for PLA homopolymers; each extracted ion chromatogram is scaled to the highest peak, giving only qualitative information when compared with all other chromatograms.

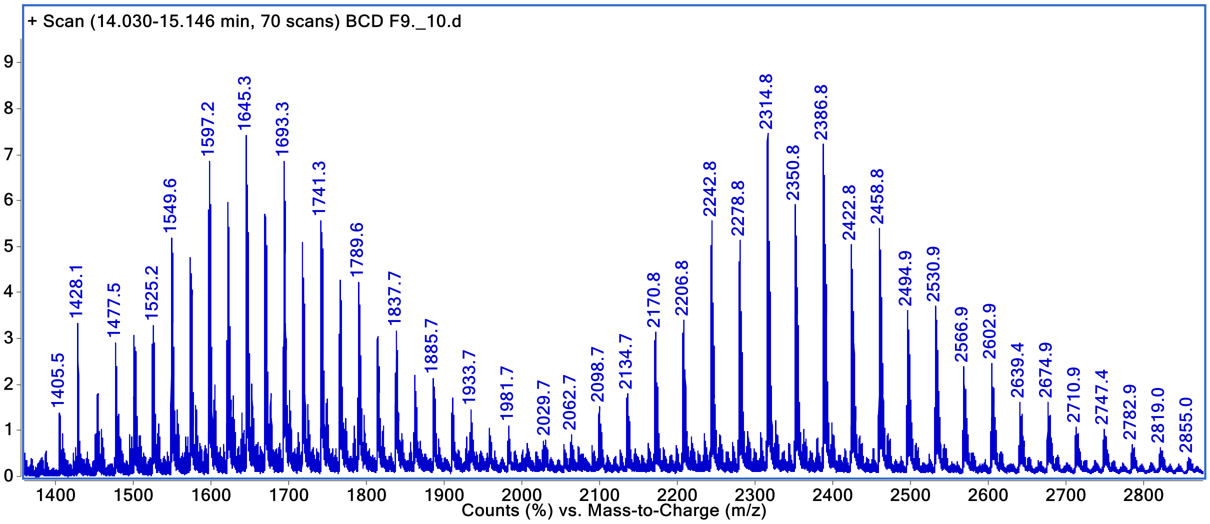

Figure S23: LC–ESI–MS spectrum β-CD-LA collected between 14 and 15 min; contains double charged (left) and triple charged (right) species of β-CD-LA (average mass of this fraction is close to 5000 Da).

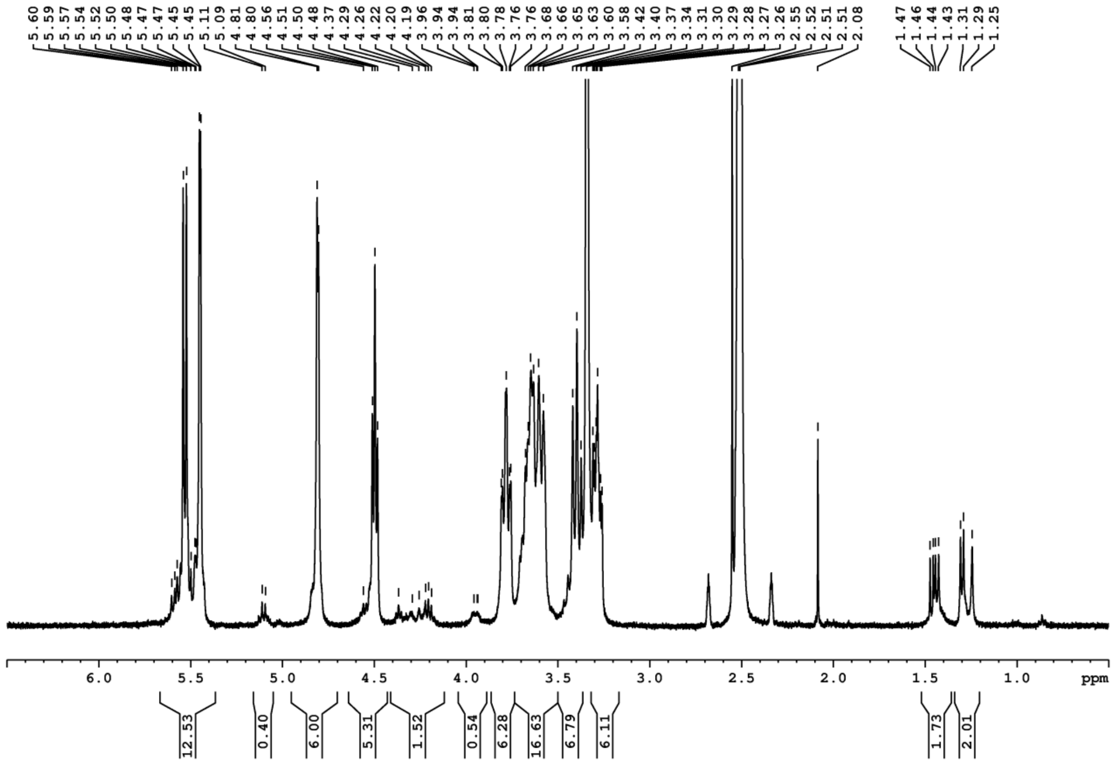

Figure S24: ^{1}H NMR spectrum of α-CD-LA F1 fraction.

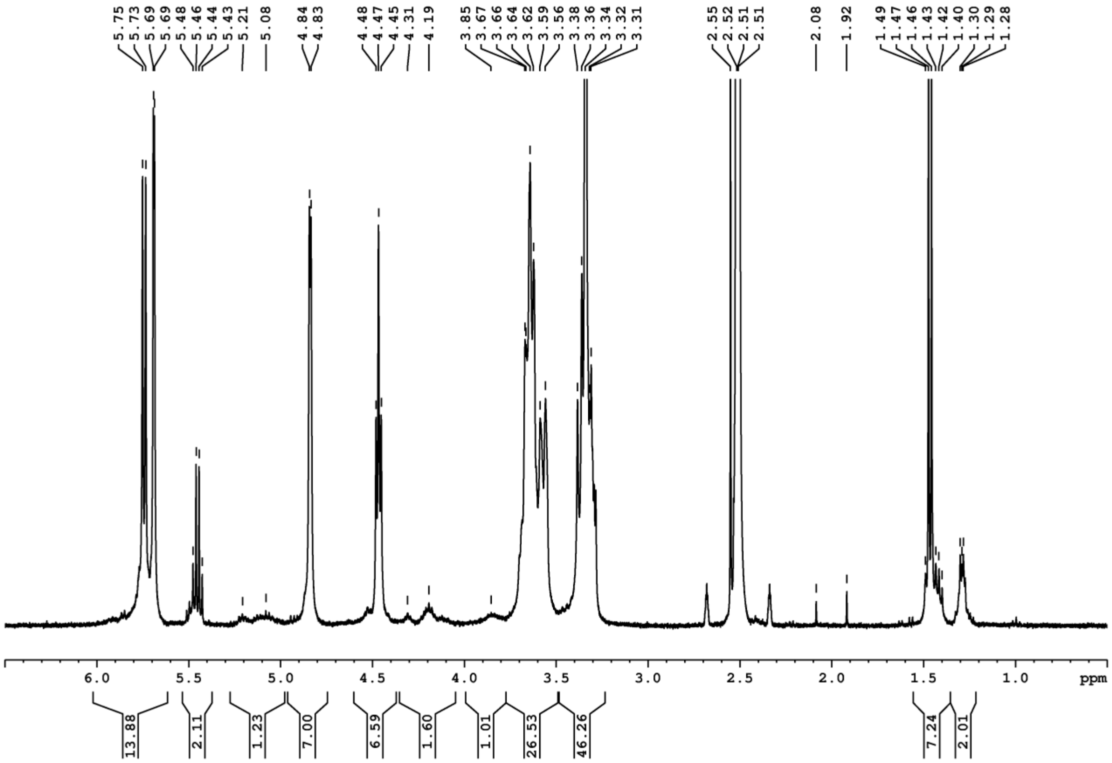

Figure S25: ^{1}H NMR spectrum of β-CD-LA F1 fraction.

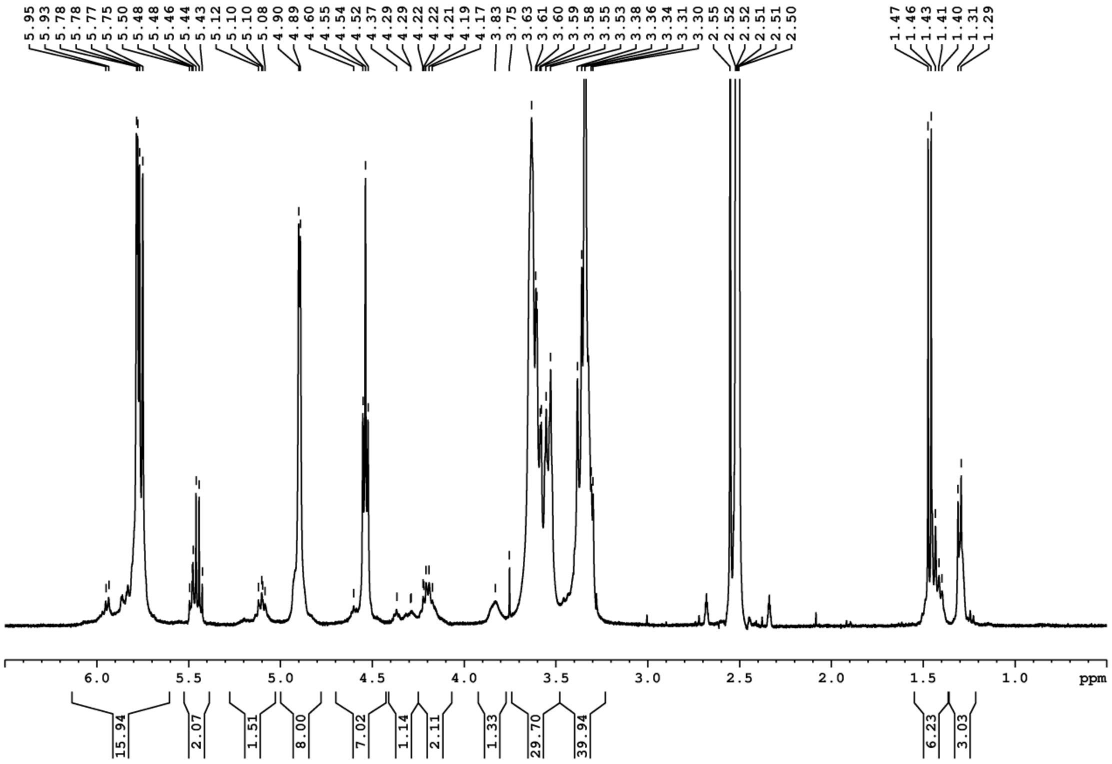

Figure S26: ^{1}H NMR spectrum of γ-CD-LA F1 fraction.

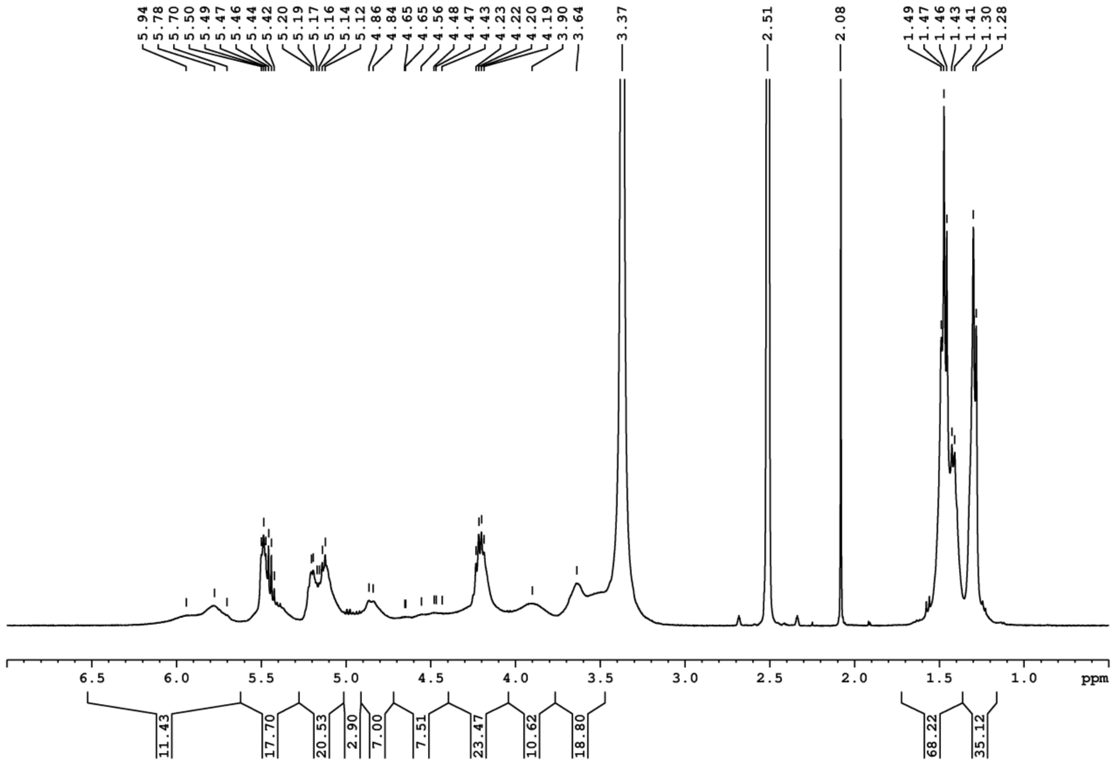

Figure S27: ^{1}H NMR spectrum of β-CD-LA F2 fraction.

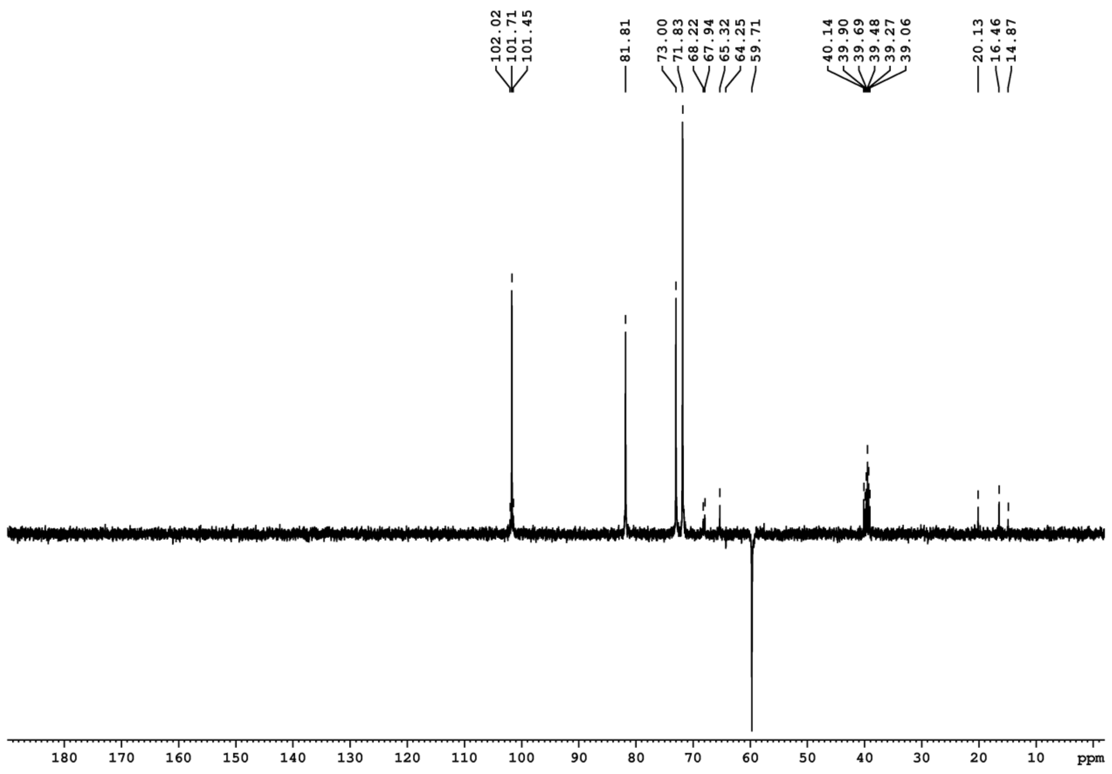

Figure S28: DEPT135-NMR spectrum of α-CD-LA F1 fraction.

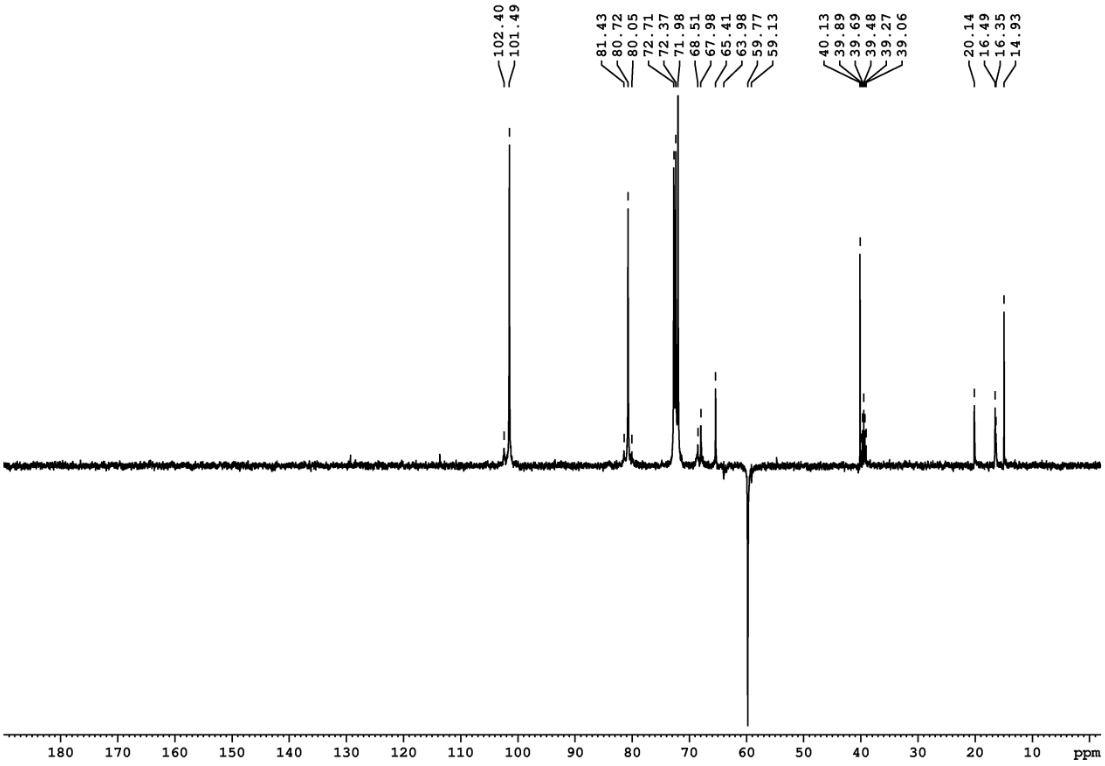

Figure S29: DEPT135-NMR spectrum of γ-CD-LA F1 fraction.

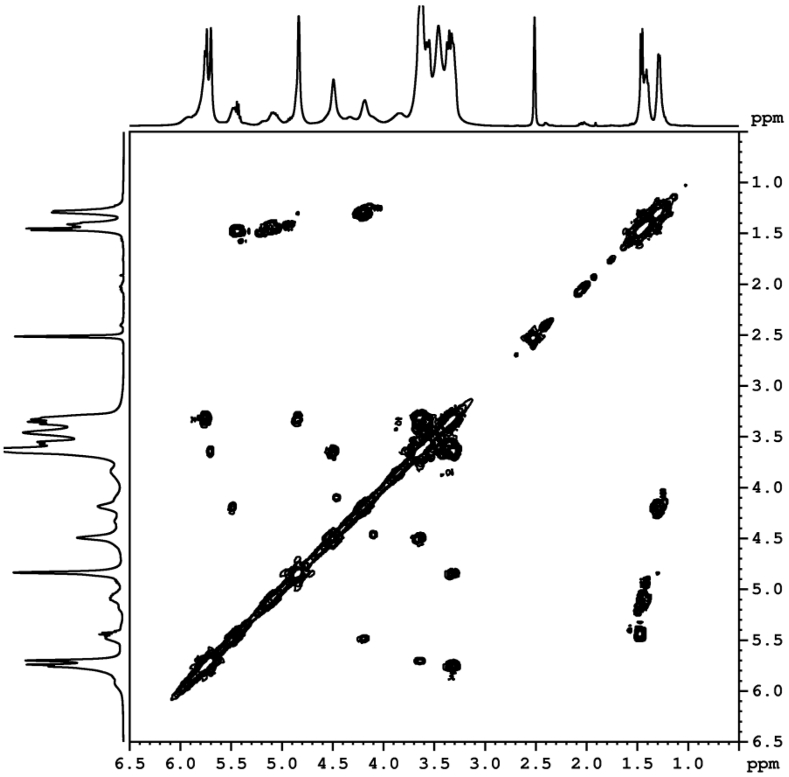

Figure S30: 2D-COSY-NMR spectrum of β-CD-LA F1 fraction.

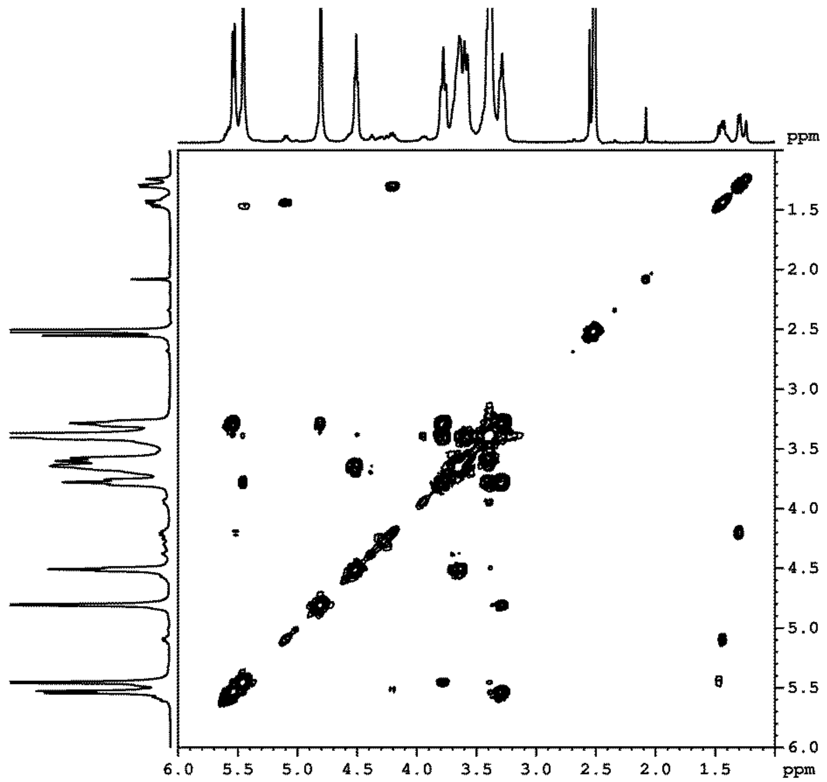

Figure S31: 2D-COSY-NMR spectrum of α-CD-LA F1 fraction.

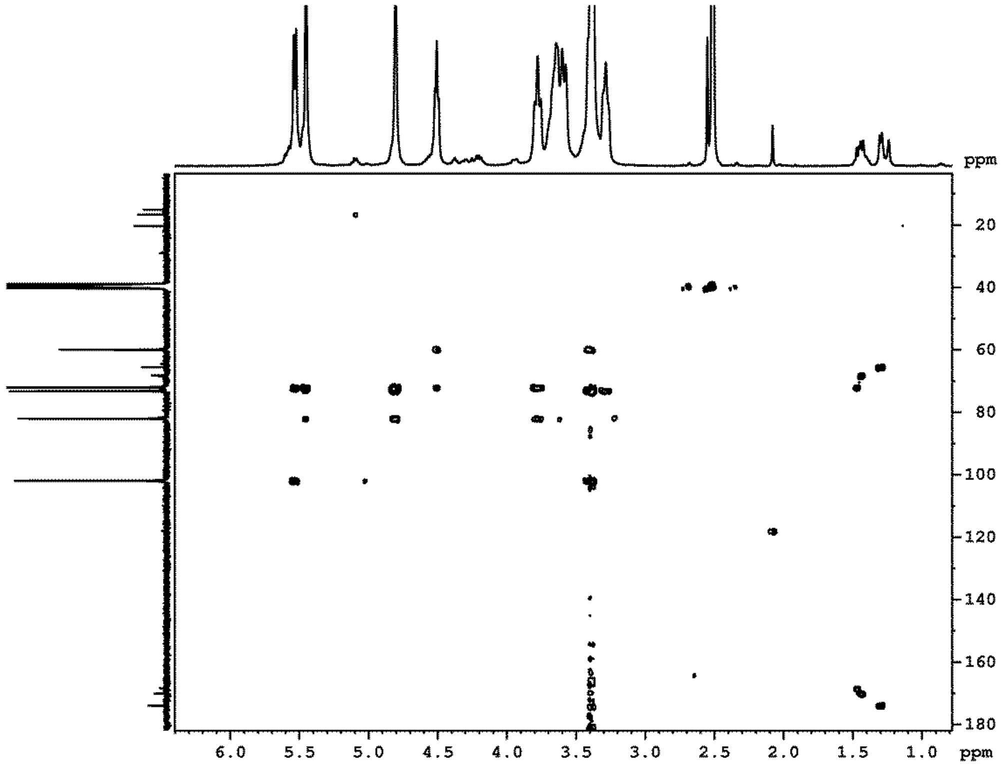

Figure S32: 2D-HMBC-NMR spectrum of α-CD-LA F1 fraction.

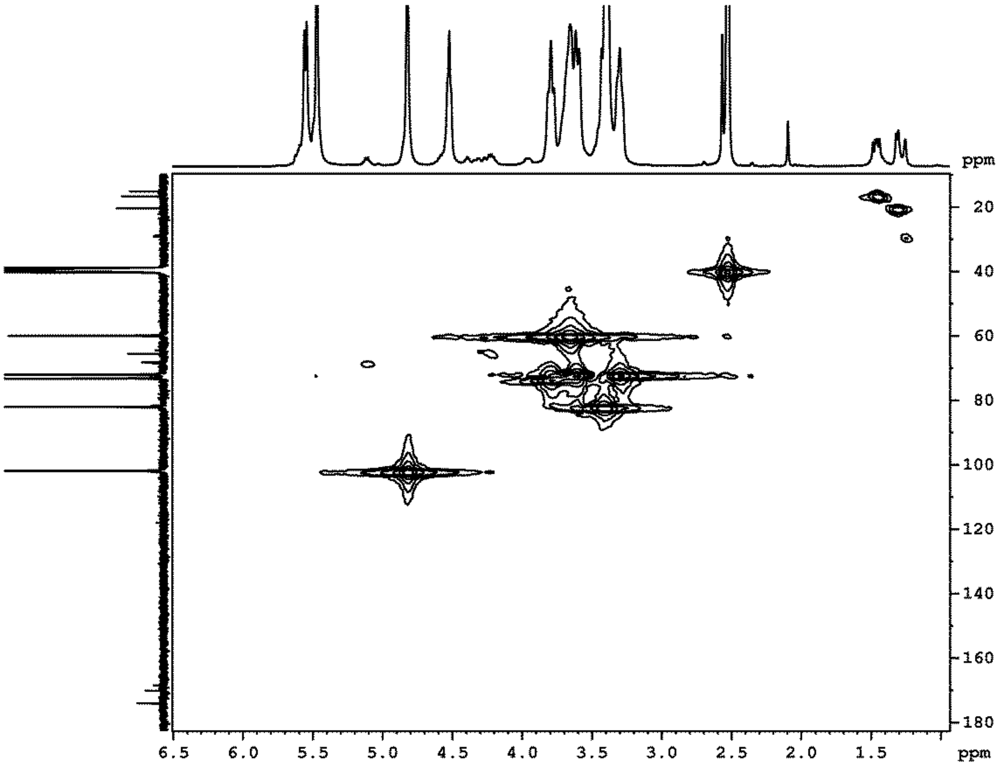

Figure S33: 2D-HMQC-NMR spectrum of α-CD-LA F1 fraction.

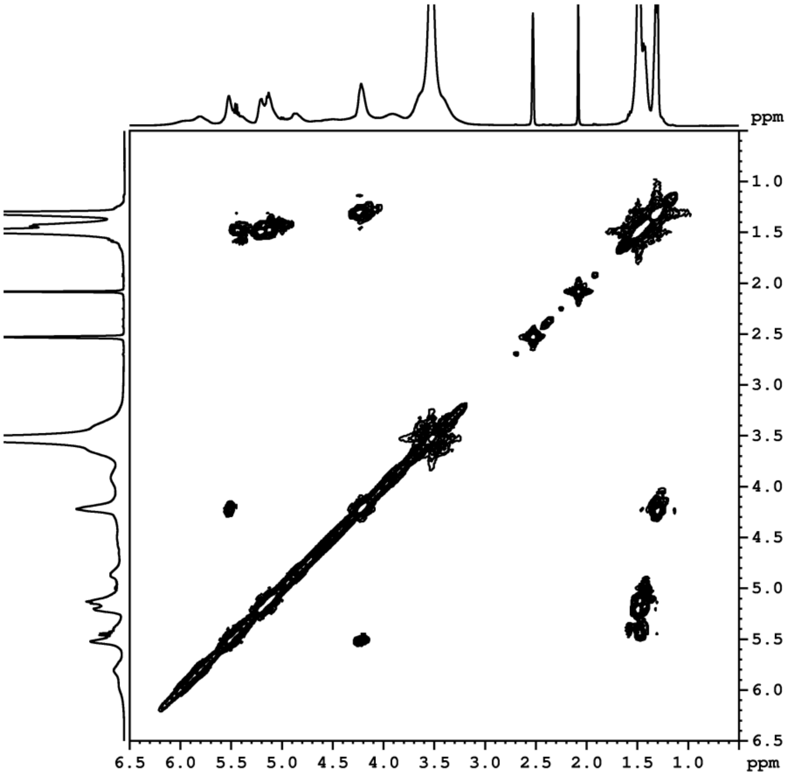

Figure S34: 2D-COSY-NMR spectrum of β-CD-LA F2 fraction.

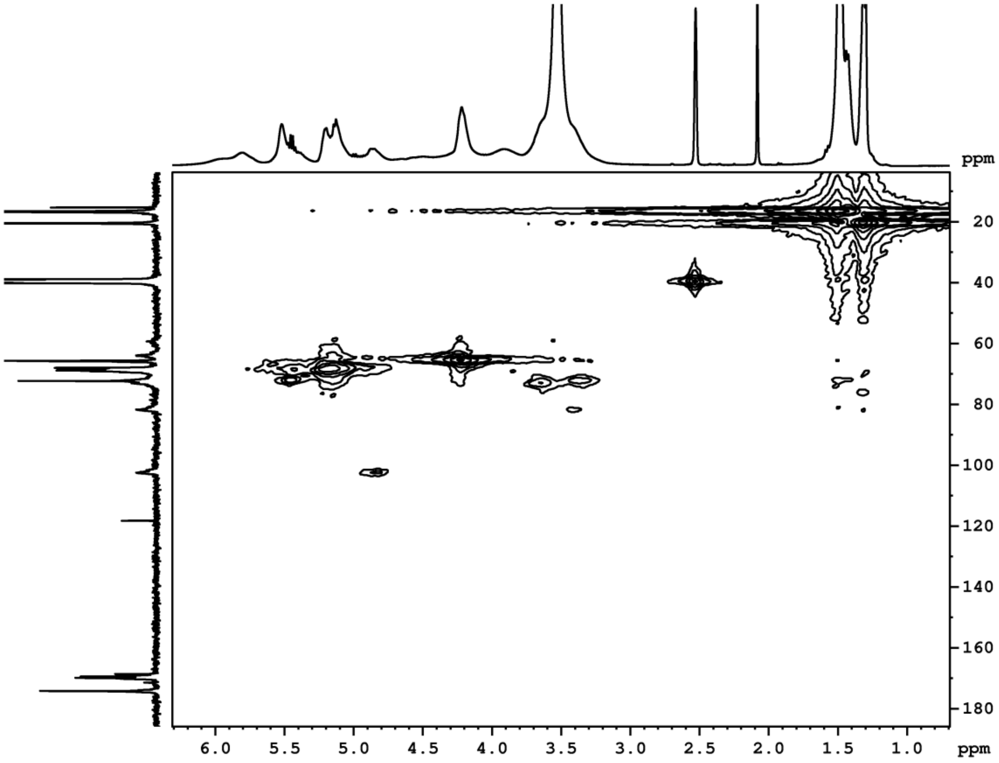

Figure S35: 2D-HMQC-NMR spectrum of β-CD-LA F2 fraction.

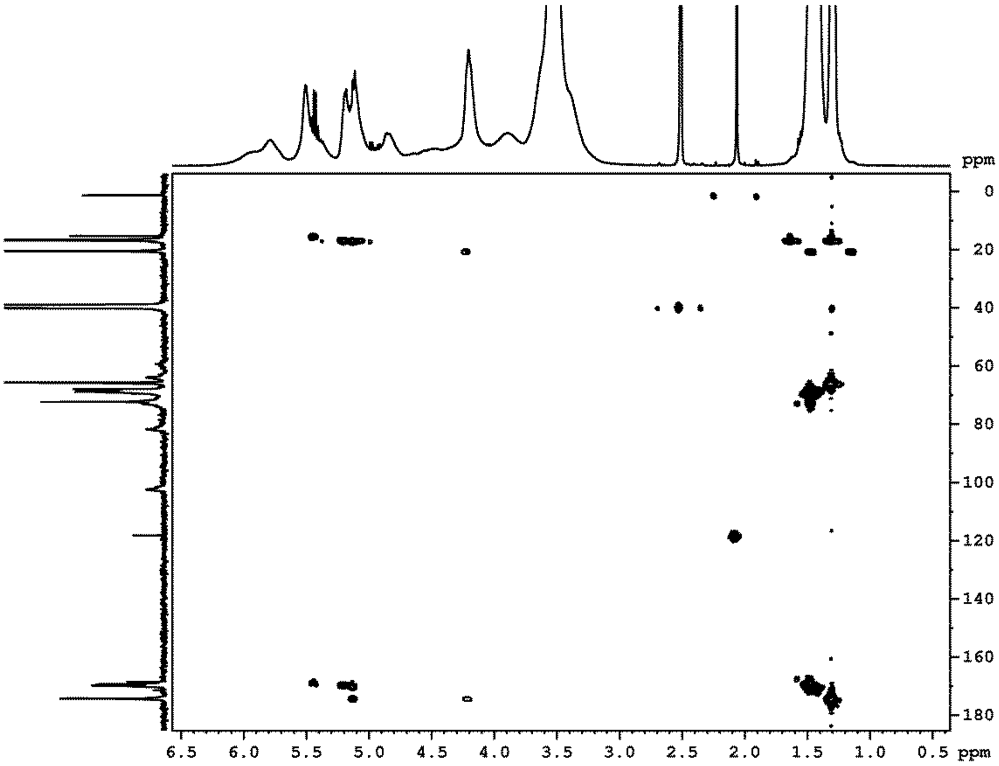

Figure S36: 2D-HMBC-NMR spectrum of β-CD-LA F2 fraction.

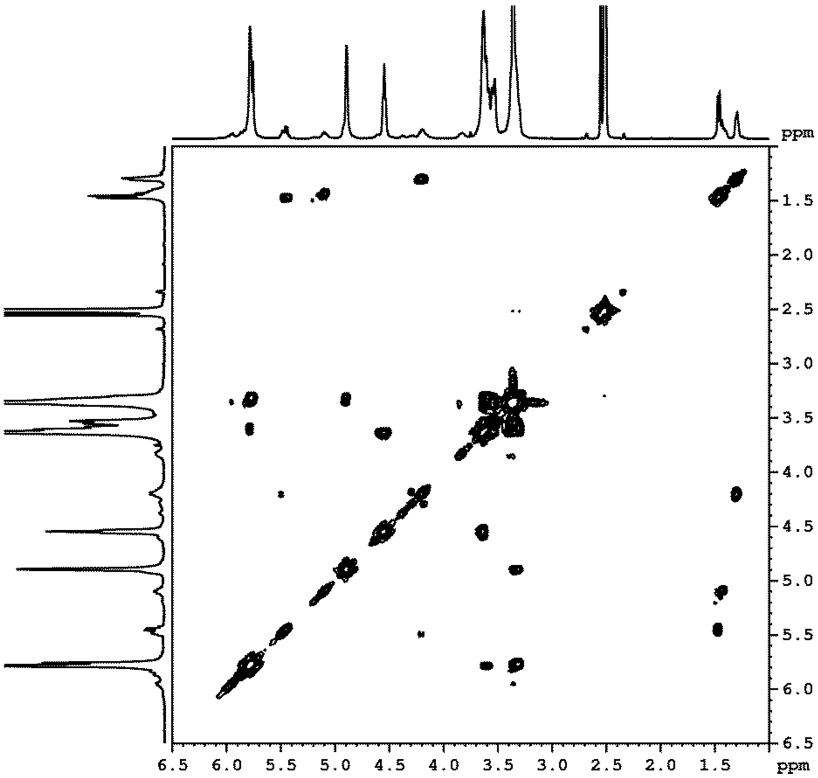

Figure S37: 2D-COSY-NMR spectrum of γ-CD-LA F1 fraction.

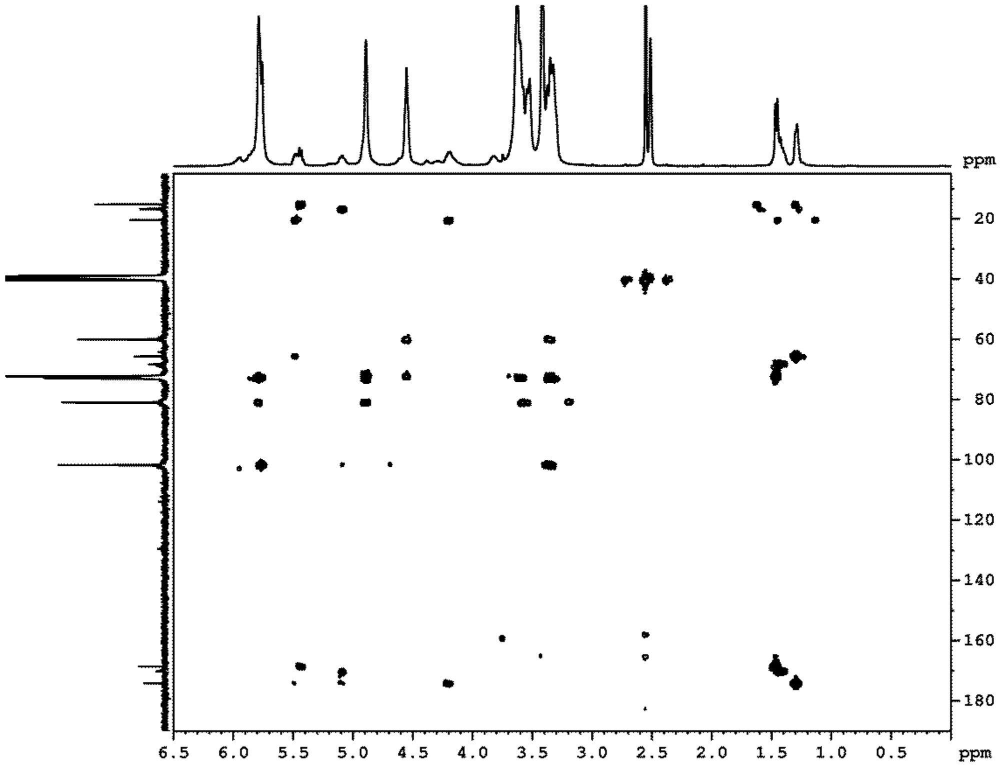

Figure S38: 2D-HMBC-NMR spectrum of γ-CD-LA F1 fraction.

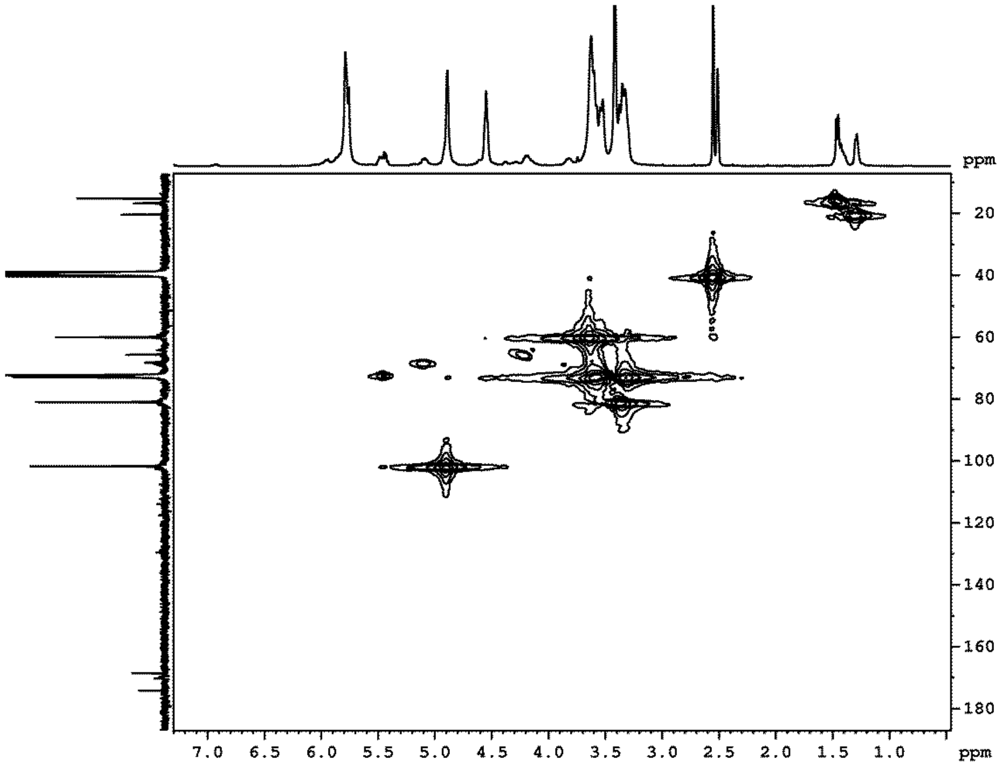

Figure S39: 2D-HMQC-NMR spectrum of γ-CD-LA F1 fraction.

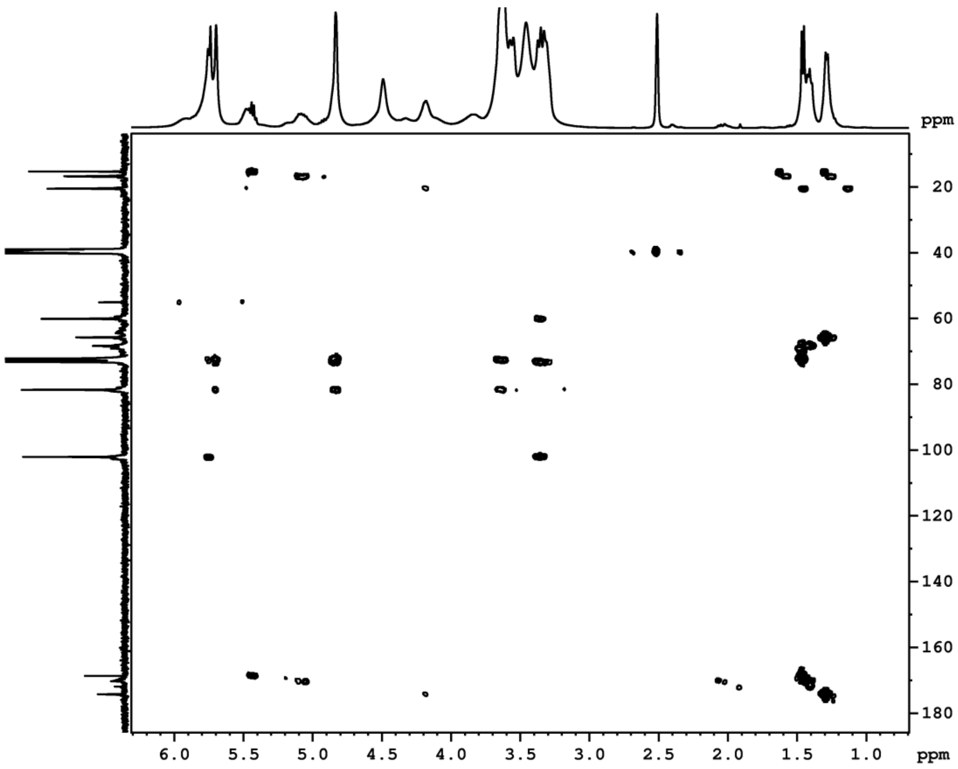

Figure S40: 2D-HMBC-NMR spectrum of γ-CD-LA F1 fraction.

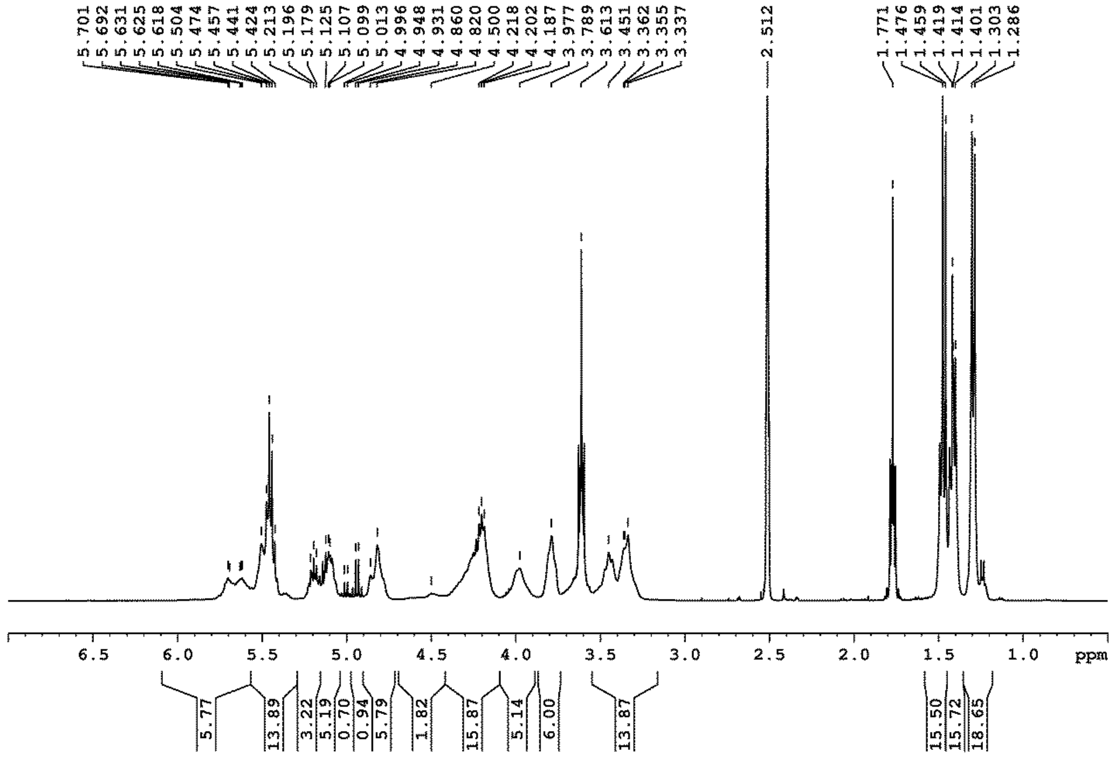

Figure S41: ^{1}H NMR spectrum of α-CD-LA F2 fraction.

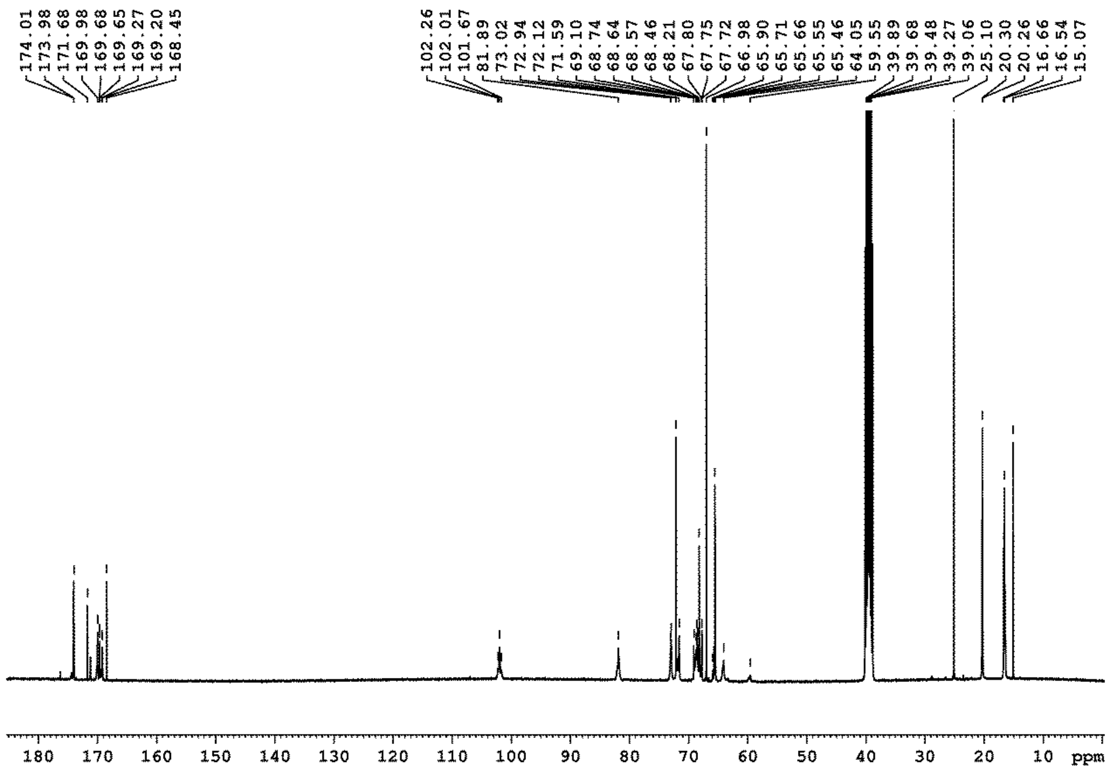

Figure S42: ^{13}C NMR spectrum of α-CD-LA F2 fraction.

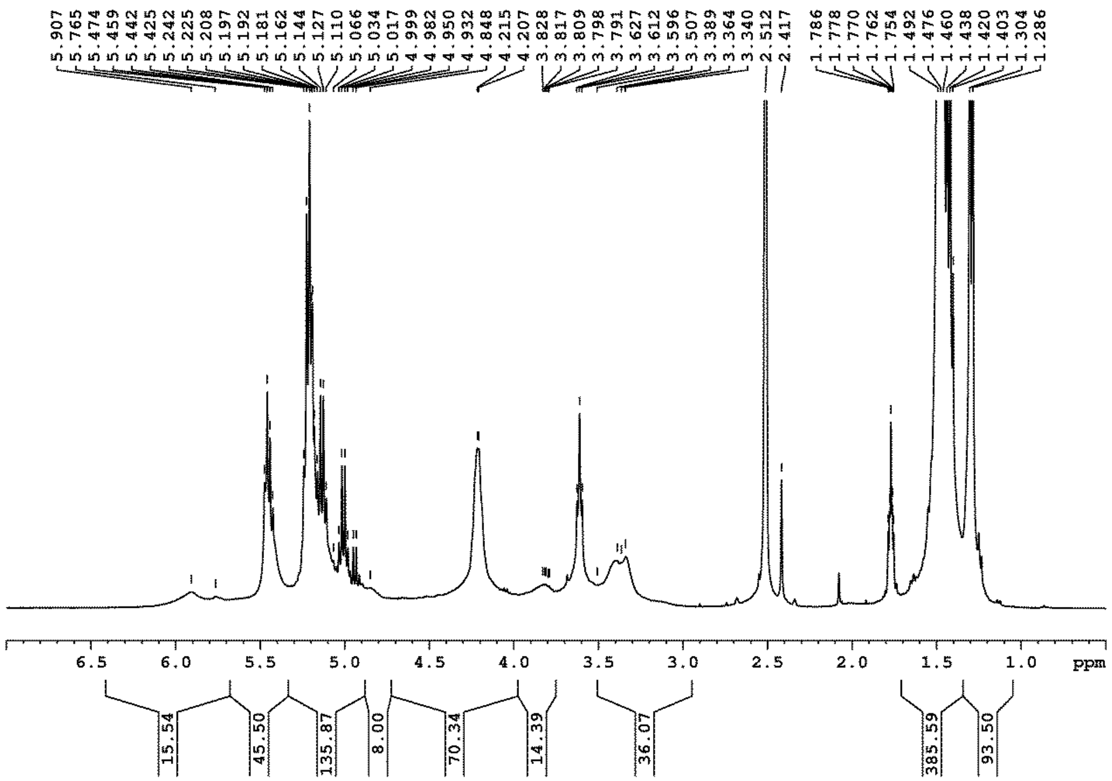

Figure S43: ^{1}H NMR spectrum of γ-CD-LA F2 fraction.

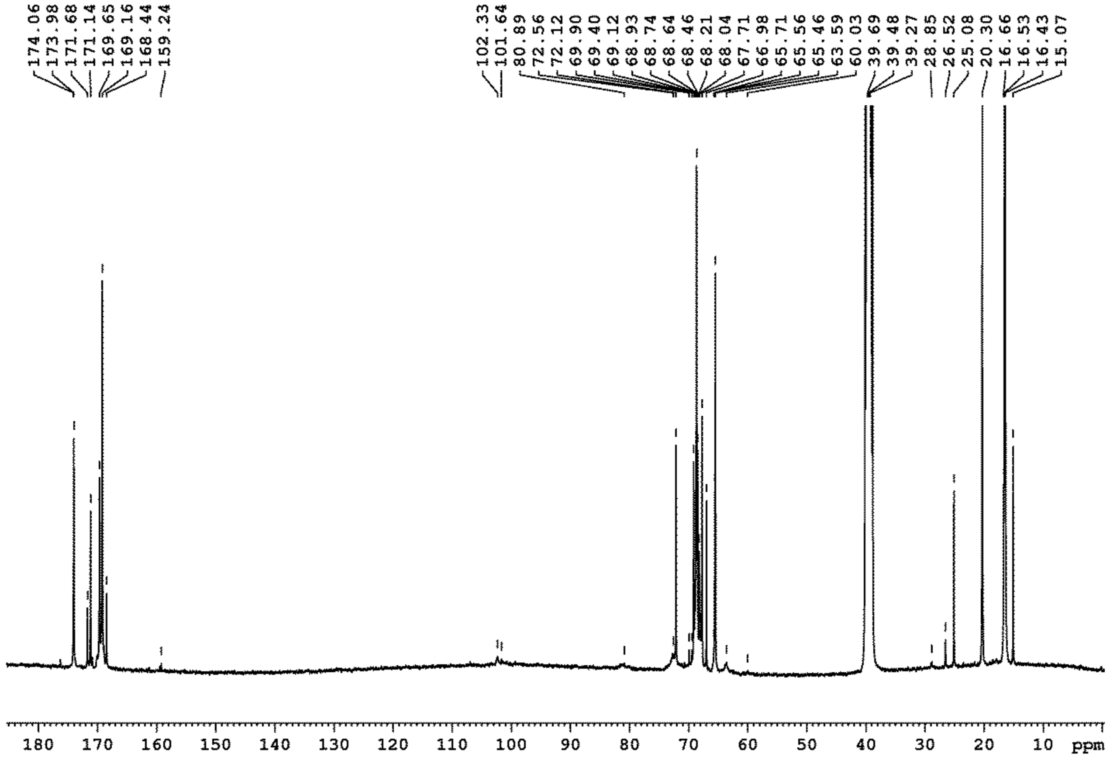

Figure S44: ^{13}C NMR spectrum of γ-CD-LA F2 fraction.
